# Evaluation of the Pediatric Regional Anesthesia Time‐Out Checklist: A Simulation Study

**DOI:** 10.1111/pan.15069

**Published:** 2025-01-24

**Authors:** Anna Clebone, Brian Duggar, Tessa N. Mandler, Barbara K. Burian, Melissa M. Masaracchia, David Polaner

**Affiliations:** ^1^ Department of Anesthesia and Critical Care University of Chicago Chicago Illinois USA; ^2^ Department of Anesthesiology, Children's Hospital Colorado University of Colorado School of Medicine Aurora Colorado USA; ^3^ Human Systems Integration Division NASA Ames Research Center Moffett Field California USA; ^4^ Northwell, Zucker School of Medicine at Hofstra New Hyde Park New York USA; ^5^ Cohen Children's Medical Center New Hyde Park New York USA; ^6^ University of Washington School of Medicine Seattle Washington USA; ^7^ Seattle Children's Hospital Seattle Washington USA

## Abstract

**Introduction:**

The Society for Pediatric Anesthesia Quality and Safety Committee developed the Pediatric Regional Anesthesia Time‐Out Checklist, consisting of 14 safety items intended to be reviewed by an anesthesia team prior to a regional anesthetic. Primarily, we hypothesized that use of this Checklist would increase the number of safety items performed compared with no checklist, evaluating the usefulness of this tool. Secondarily, we hypothesized that, after checklist training, subjects would show better clinical judgment by electing to perform a regional anesthetic in scenarios in which no programmed error existed and electing to not perform a regional anesthetic in scenarios in which a programmed error did exist.

**Methods:**

Each anesthesia attending/trainee pair participated in 12 different randomized video‐recorded medium‐fidelity regional anesthesia simulation scenarios, receiving checklist training after half of the scenarios had been completed by each pair. In four of the scenarios, subjects were expected to decline to perform the regional anesthetic because of an error programmed into the scenario. Two errors consisted of a maximum dose of local anesthetic given by the surgeon immediately prior to the planned regional anesthetic and two errors consisted of coagulation issues prior to neuraxial block (1 with a low platelet count and 1 receiving low molecular weight heparin). Scenarios were scored for the number of safety items identified and performed by the subjects. Additionally, the team's choice to perform the regional anesthetic or abort was recorded.

**Results:**

One‐hundred and thirty‐two scenarios were performed by 22 physicians. A greater number of safety items were completed after training on the Pediatric Regional Anesthesia Time‐Out Checklist, for each of 11 individual groups and when data from all groups was pooled, *p* < 0.001, 95% CI (0.33, 0.41). Overall, 78% of safety items studied were performed after checklist training compared to 41% of safety items performed prior to training. The team's choice to perform or abort the regional anesthetic occurred as expected more often (92% of scenarios) after Checklist training, compared to before checklist training (77% of scenarios), *t* = 3.41; *p* = 0.001, 95% CI (0.03, 0.27). Teams chose to perform the regional anesthetic despite a programmed error in three scenarios (0.05%) prior to Checklist training and no scenarios (0%) after Checklist training.

**Conclusion:**

Pediatric Regional Anesthesia Time‐Out Checklist training led to an increased number of safety items performed prior to a simulated anesthetic.

## 1 Introduction

**FIGURE 1:**
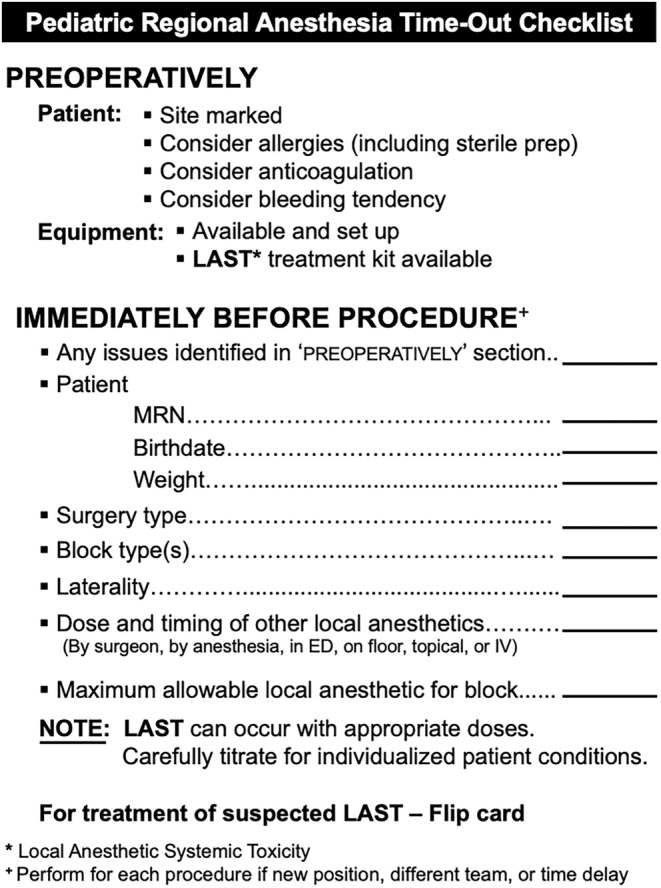
Pediatric Regional Anesthesia Time‐Out Checklist.

Checklists in the perioperative environment are intended to improve safety and decrease errors. These checklists are typically developed using expert consensus and evidence‐based literature. The Regional Anesthesia Time‐Out Checklist was developed by the Society for Pediatric Anesthesia Quality and Safety Committee in 2016 (Figure 1). It contains the 14 items that experts determined, by consensus, should be reviewed prior to performing a regional anesthetic and is intended to create a shared mental model for the operating room team prior to performing a regional anesthetic [1]. The use of this checklist in actual practice is intended to meet four major goals: (1) standardize the review of important information and preparation for the administration of regional anesthesia, (2) facilitate the development of a shared mental model among surgical room team members, (3) avoid the occurrence of local anesthetic systemic toxicity (LAST), and (4) avoid wrong‐sided block [1]. The rate of complications in pediatric regional anesthetics is < 1 in 100 000 [2], but decreasing this rate even further is an important goal, as any error may be catastrophic. Performing all safety checks immediately prior to a regional anesthetic could improve quality and decrease medical errors [1].

A crucial part of checklist development is evaluation and testing in simulation. For our primary outcome, we tested the hypothesis that the Regional Anesthesia Time‐Out Checklist would increase the number of safety items performed by 11 attending and resident dyads during 132 simulated scenarios (Table 1). For our secondary outcomes, we hypothesized that after checklist training subjects would show better clinical judgment by electing to perform a regional anesthetic in scenarios in which no programmed error existed and electing to not perform a regional anesthetic in scenarios in which a programmed error did exist, and that participants would rate this Checklist highly.

**TABLE 1:** Safety items to be reviewed prior to performing a pediatric regional anesthetic.

Site marked
Consider allergies (including sterile prep)
Consider anticoagulation
Consider bleeding tendency
Equipment available and set up
LAST treatment kit available
MRN
Birthdate
Weight
Surgery type
Block type(s)
Laterality
Dose and timing of other local anesthetics
Maximum allowable local anesthetic for block

## 2 Methods

### 2.1 Scenario Development

A query was performed of the Pediatric Regional Anesthesia Network (PRAN) database, the Wake‐Up‐Safe database, and the anesthesia closed‐claims database for relevant cases, and a PubMed search was performed for relevant cases in the literature from April 2007 to January 2016. These cases were then classified by type of error. Whenever possible, scenarios were adapted from these cases.

Twelve distinct study scenarios in total were developed, encompassing two groups of six scenarios. Each group of six scenarios corresponded with the demographics (ages, American Society of Anesthesiologists physical status, emergency status, and weight) [3], and block type [3] of patients in the PRAN database [2] query. Patient identifiers and details used in the study were fictitious.

Each scenario stem provided the following types of information related to a pediatric case involving the administration of regional anesthesia:

- patient name, age, weight (kg), medical record number (MRN), and birthdate;
- allergies (including sterile prep);
- type of surgery to be performed or just performed;
- the type of block planned; and
- the types, doses, administration method, and timing of any anesthetics already administered by a surgeon and/or an anesthesiologist.

In four of the scenarios, subjects were expected to decline to perform the regional anesthetic because of an error programmed into the scenario. Two errors consisted of a maximum dose of local anesthetic given by the surgeon immediately prior to the planned regional anesthetic and two errors consisted of coagulation issues prior to neuraxial block (1 with a low platelet count and 1 receiving low molecular weight heparin).

### 2.2 Scenario Randomization

Twelve scenarios were performed by each group. The placement of the scenarios was intentional within a planned framework to avoid having two scenarios with programmed errors in a row and to avoid having the subjects begin the study with a scenario containing a programmed error. The framework contained the following rules: Slots 3, 5, 9, and 11 were reserved for the four scenarios containing programmed errors. Slots 1, 2, 4, 6, 7, 8, 10, and 12 were reserved for the four scenarios which did not contain programmed errors.

Before starting the study, for each of the 11 groups, the following two‐part randomization procedure took place using www.randomizer.org: (1) The four scenarios with programmed errors were randomized into slots 3, 5, 9, and 11. Randomization was performed so that the first half of the study (6 scenarios) prior to checklist training contained a scenario with one programmed error involving local anesthetic overdose and one programmed error involving coagulopathy, and the second half of the study after checklist training (6 scenarios) contained one potential error involving local anesthetic overdose and one potential error involving coagulopathy. (2) Scenarios without programmed errors were randomized to slots 1, 2, 4, 6, 7, 8, 10, and 12.

### 2.3 Procedures

Pilot testing of these scenarios only was first completed at the University of Chicago, IRB#17‐0274. For the simulation study reported in this manuscript, approval was obtained from the University of Colorado School of Medicine Institutional Review Board, #18‐0074. Subjects were recruited from anesthesiology faculty, residents, and fellows at Children's Hospital Colorado with verbal consent for participation and written consent for video recording. Inclusion criteria were previous experience performing pediatric regional anesthetics and willingness to participate. All study procedures took place in an empty anesthetizing location in a medium‐fidelity setting, with procedural equipment available, including medications and needles. The neonatal simulator did not have vital sign, auscultation, or airway functionality.

Subjects first underwent training for the simulation by participating in a practice scenario. Anesthesia attending and trainee pairs alone then participated in the first set of six scenarios. Then, they took a short break and completed a survey (Supporting Information: Survey). Next, subjects underwent training for the Regional Anesthesia Time‐Out Checklist (Figure 1) (Supporting Information: Training for Checklists), after which they participated in the second set of six scenarios. Finally, they participated in a debrief of the simulation and filled out a second survey (Supporting Information: Survey).

### 2.4 Study Scenarios

Each scenario was designed to be 2–4 min in length. For each scenario, each subject was first given a card with both the stem and scenario information specific to their role. The card given to the attending contained the stem and additional information specific for the attending and the card given to the trainee contained the same stem but a different set of additional information specific for the trainee. Then, subjects were instructed to communicate and take the necessary steps that they normally would to perform a regional anesthetic. Subjects were not instructed on which member of the team should initiate a time‐out procedure. Subjects were told that each scenario would end at the time at which the block needle would be inserted into the patient.

### 2.5 Data Evaluation

The video recordings were reviewed, and safety items achieved (Table 1) and the team's choice to perform the regional anesthetic or abort was recorded by two investigators (A.C. and T.M.). Each of the 15 safety items (Table 1) was marked by the rater as a score of 0 if not performed, 0.5 if incompletely performed, or 1 if completely performed. Next, these ratings were discussed, and any discrepancies in ratings were resolved. This process was repeated until agreement was achieved, which occurred after watching approximately 1/10th of the total scenarios. After the two investigators were satisfied that they had achieved unanimity on the scoring criteria, the remainder of the videos were scored by one investigator (T.M.).

### 2.6 Sample Size

The necessary number of scenarios to test for the primary outcome was calculated. This primary outcome represents a re‐analysis of existing data. A paired *t*‐test showed that at least 10 groups would be needed, with a mean difference of 0.5, standard deviation of differences of 0.5; power = 0.8; and significance level = 0.05 (without Bonferroni adjustment). The power and significance level are similar to that used for several previous cognitive aid design evaluation studies [4, 5, 6].

### 2.7 Statistics

Each of the 14 action items on the Pediatric Regional Anesthesia Time‐Out Checklist (Figure 1), such as “Site marked,” and “Consider allergies (including sterile prep),” was considered a “safety item” for our analysis.

Data were analyzed using the GIGA calculator, the Social Science Statistics Calculator, and GraphPad. The Shapiro–Wilk test was used to determine the distribution of data. A two‐tailed Wilcoxon Signed‐Rank test was used to compare the number of safety items performed by each group before and after training. A paired *t*‐test was used to compare the total number of safety items performed before and after training for all groups when these data were pooled. A Bonferroni correction for multiple analyses revealed that *p* < 0.004 should be considered significant. A Mann Whitney *U* test was used to determine if differences between attendings and trainees existed with regards to their baseline views on checklists.

## 3 Results

One‐hundred and thirty‐two scenarios were performed by 22 anesthesiologists. All attending physicians were board certified and pediatric fellowship trained, with 9 [3, 11] median [IQR] years of experience, age 43.9 (9.6) mean (SD), eight male, three female. Trainees were eight Pediatric Anesthesiology fellows, and three residents (2 CA‐2 and 1 CA‐3), age 31.7 (1.7) mean (SD), seven male, four female. All subjects had performed or administered multiple blocks per month and had at least 2 years of experience performing pediatric regional anesthetics except one trainee who was without this experience (Supporting Information: Survey Results).

For our primary outcome, we found that a greater number of safety items were completed after training on the Pediatric Regional Anesthesia Time‐Out Checklist, for each of 11 individual groups (*p* = 0.002) and when data from all groups were pooled (*p* < 0.001) (Table 2). In total, 78% of safety items studied were performed after training, and 41% of safety items were performed prior to training.

**TABLE 2:** Number of safety items completed prior to and after training on the Regional Anesthesia Time‐Out Checklist.

Group	Prior to training: No. of safety items completed; median [interquartile range]	After training: No. of safety items completed; median [interquartile range]	*Z*	*p* ^a^	95% CI		
1	31.5; 0 [0, 0–1]	63; 1 [0.5–1]	−4.50	< 0.001	0.24, 0.51		
2	46; 1 [0–1]	62; 1 [0–1]	−3.04	0.002	0.05, 0.33		
3	32; 0 [0–1]	62; 1 [0–1]	−4.36	< 0.001	0.24, 0.59		
4	39; 0.5 [0–1]	62; 1 [0–1]	−3.82	< 0.001	0.14, 0.41		
5	36.5; 0 [0–1]	62; 1 [0–1]	−3.98	< 0.001	0.16, 0.45		
6	30; 0 [0–1]	72.5; 1 [1–1]	−5.37	< 0.001	0.38, 0.63		
7	46.5; 1 [0–1]	64; 1 [0–1]	−3.17	0.002	0.07, 0.35		
8	31.5; 0 [0–1]	61; 1 [0–1]	−4.17	< 0.001	0.21, 0.49		
9	35.5; 0 [0–1]	72; 1 [1–1]	−5.12	< 0.001	0.31, 0.56		
10	32; 0 [0–1]	64.5; 1 [1–1]	−4.79	< 0.001	0.25, 0.52		
11	18.5; 0 [0–0.5]	73; 1 [1–1]	−6.08	< 0.001	0.55, 0.77		

Variable	Prior to training: No. of safety items completed (mean, SD)	After training: No. of safety items completed (mean, SD)	*t*	*p* ^b^	95% CI	df	Standard error of difference
Total Safety Items Performed^c^	379 (0.42, 0.48)	718 (0.78, 0.414)	17.59	< 0.001	0.33, 0.41	1844	0.021

*Note*: Partially completed safety items were scored as 0.5; fully completed items were scored as 1.

^a^ For each individual group, a two‐Tailed Wilcoxon Signed‐Rank Test was used for non‐ normally distributed data.

^b^ For the “Total Safety Items Performed,” a paired two‐tailed *t*‐test was used.

^c^ 923 total safety items possible prior to training, and 923 total safety items possible after training.

For our secondary outcome, we found that the team's choice to perform or abort the regional anesthetic occurred as expected more often after Checklist training, *t* = 3.41; *p* = 0.001 (Table 3). Additionally, prior to Checklist training, in three scenarios, teams chose to perform the regional anesthetic despite a programmed error. Two of these scenarios were programmed to contain the error “neuraxial with a low platelet count” and in one the surgeon had given the maximum dose of local anesthetic immediately prior. After Checklist training, no teams chose to perform the regional anesthetic in scenarios with a programmed error.

**TABLE 3:** Pediatric Regional Anesthesia Time‐Out Checklist and regional anesthetic performance and non‐performance.^a^

Variable	Prior to Checklist training	After Checklist training	Total number	Total %	*t* ^b^	*p*	95% CI
Total scenarios	66	66	132				
Scenarios containing a programmed error	22	22	44				
Scenarios without a programmed error	44	44	88				
Met the expectation for performing the block	51	61	112	85	−3.41	0.001	0.03, 0.27
Did NOT meet the expectation for performing the block^c^	15	5	20				
Performed the block in a scenario containing a programmed error	3	0			−1.75	0.08	−0.0052, 0.096
Did NOT perform the block in a scenario containing a programmed error	63	66					

^a^ Subjects were asked to not perform the block in scenarios in which they detected a prohibitive safety issue.

^b^ Paired *t*‐test for dependent means.

^c^ Includes if they did not clearly indicate if they were performing the block, if they were expected to perform the block and did not, and if they were expected to not perform the block and did.

Overall, trainees rated the Checklist favorably on multiple metrics of usability and design (Table 4 and Supporting Information). Trainees and attendings did not differ on their baseline views about checklist efficacy overall (Table 5). In general, both trainees and attendings had a high amount of training and experience with checklists and thought favorably of the role of checklists in the medical setting (Table 4).

**TABLE 4:** Subject Opinions on the Regional Anesthesia Time‐Out Checklist.

Question	Attending median [interquartile range]	Trainee median [interquartile range]
It was easy to read the checklist^a^	5 [5, 5]	5 [5, 5]
Overall, the checklist is too long^a^	2 [2, 3]	2 [2, 3]
I clearly understood what I was supposed to do/think about relative to items in the PREOPERATIVE section of the checklist^a^	4 [4, 5]	5 [4, 5]
The PREOPERATIVE section contains all the steps that are essential^a^	4.5 [4, 5]	4 [4, 5]
I clearly understood what I was supposed to do relative to items in the IMMEDIATELY BEFORE PROCEDURE section of the checklist^a^	4 [4, 5]	5 [3, 5]
The IMMEDIATELY BEFORE PROCEDURE section contains all the steps that are essential^a^	4 [4, 5]	4 [4, 5]

^a^ Likert scale: 1 = Strongly Disagree; 2 = Disagree; 3 = Neutral; 4 = Agree; 5 = Strongly Agree.

**TABLE 5:** Survey data—General opinions on checklists.

Question	Attending median [interquartile range]	Trainee median [interquartile range]	*U*	*Z*	*p* ^c^	95% CI
How much training have you previously had regarding the use of checklists as a part of your normal practice (e.g., the time‐out checklist or WHO surgical safety checklist)?^a^	4 [3, 5]	4 [4, 5]	50	−0.66	0.509	−0.56, 0.92
How much experience do you have using checklists as a part of your normal practice (e.g., the time‐out checklist or WHO surgical safety checklist)?^a^	4 [4, 5]	5 [4, 5]	53.5	−0.43	0.667	−0.62, 0.98
For normal practice, I do not think checklists are very useful^b^	0 [1, 2]	0 [1, 2]	47.5	0.82	0.412	−0.85, 0.30
A normal checklist for administering regional anesthetics is generally unnecessary^b^	2 [2, 2]	2 [1, 2]	55.5	−0.30	0.764	−0.58, 0.40

^a^ Likert scale: 1 = None; 5 = Great Deal.

^b^ Likert scale: 1 = Strongly Disagree; 2 = Disagree; 3 = Neutral; 4 = Agree; 5 = Strongly Agree.

^c^ Mann Whitney *U* test. The critical value of *U* at *p* < 0.05 is 30.

## 4 Discussion

Use of the Pediatric Regional Anesthesia Time‐Out Checklist increased the number of safety items performed by teams of an Anesthesiology Attending–Trainee pair prior to performing a simulated regional anesthetic. This result was found across a wide variety of scenarios involving a range of blocks performed in clinical practice; the 12 distinct scenarios were designed to encompass types of errors previously reported in the literature. We also found that use of this Checklist increased the number of safety items performed by teams including attendings and trainees across multiple levels of prior experience.

Unexpectedly, only 78% of safety items were performed after training. This may have been because some items were considered irrelevant to a particular block. For example, laterality is irrelevant for a neuraxial block. Safety items may also not have been completed if the team decided that the block was unsafe and therefore aborted the procedure before all safety items could be considered.

After Checklist training, the team's choice to perform or abort the regional anesthetic met expectations more frequently. In three scenarios prior to checklist training, teams chose to perform the regional anesthetic despite a programmed error. After Checklist training, no teams chose to perform the regional anesthetic in scenarios with a programmed error. Even though this difference was just short of statistical significance, we feel that it is clinically important because the consequences of performing a block that could result in LAST or neuraxial bleeding could be catastrophic.

Subjects also rated the Pediatric Regional Anesthesia Time‐Out Checklist highly in a post‐study survey. Specifically, subjects agreed that the checklist was easy to read and understand, and generally thought that the length was appropriate and that essential steps were included. Subjects also liked the order of the safety items on the checklist.

### 4.1 Context

Our results align with previous research on checklist efficacy. In one study, when a trainee was asked to manage LAST alone, they performed twice as many critical tasks when using the American Society of Regional Anesthesia and Pain Medicine checklist compared with no checklist [7]. Our study differs because we examined a Time‐Out checklist intended for use prior to a procedure, whereas that initiative examined a critical event checklist used to guide therapeutic management in a crisis situation. Additionally, we used pairs of anesthesiologists as subjects, an accurate replication of many situations during which regional anesthetics are performed in academic institutions. All subjects agreed that the Checklist was good for promoting positive team interactions and establishing a shared mental model. Across groups, the number of times that each pairing had worked together previously varied, showing the potential utility of the Checklist for teams of varying levels of interpersonal familiarity. We believe that the Pediatric Regional Anesthesia Time‐Out Checklist would be useful for a variety of teams. For example, in private practice settings the Checklist could be performed with an anesthesiologist and circulating nurse or surgical assistant. This second individual could cross‐check the regional anesthetic safety items contained on the Checklist.

In one large retrospective study, the introduction of a Regional Anesthesia Time‐Out checklist reduced the incidence of wrong‐sided block in the adult population [8]. Our Checklist also addresses laterality, but differs in that it is intended for the pediatric population. Additionally, we did not address deployment and rollout in our paper, which are necessary components of checklist implementation.

Although serious adverse events in pediatric regional anesthesia are rare [9], potential complications may be due to preventable factors. In large database studies, regional anesthetic dose variability has been seen [10, 11], which has the potential to cause patient harm. We sought to include these avoidable factors in the design of the Pediatric Regional Anesthesia Time‐Out Checklist [1]. Incorrect dosage may account for a fifth of drug administration errors overall [12], and avoiding this error is particularly important in pediatric patients. The Pediatric Regional Anesthesia Time‐Out Checklist asks clinicians to carefully consider the dose of local anesthetic for a regional anesthetic, a recommended step for avoiding LAST [13, 14].

### 4.2 Implications

Our findings have implications for improving the safety of pediatric anesthesia. Our results suggest that following expert human factors guidance to intentionally design safety checklists is effective [15]. Standardizing safety metrics can improve patient care [16], and a pre‐procedure checklist is one such intervention. Any checklist, however, must be tailored to its domain to be useful. The Pediatric Regional Anesthesia Time‐Out Checklist was developed using expert judgment through multiple iterations.

The Pediatric Regional Anesthesia Time‐Out Checklist is divided into two sections, as described previously: [1] The “Preoperatively” section is envisioned as a “double‐check” for the clinician providing regional anesthesia. This helps to orient the clinician and provide a safety check if the anesthesiologist is supervising several regional anesthetics on different patients sequentially. For that reason, we left in safety items that may have been previously addressed by the perioperative team, for example, during the performance of the World Health Organization surgical safety checklist [17].

The second section, “Immediately Before Procedure” addresses the fact that the perioperative environment is rapidly changing, with a need for high levels of communication and situational awareness. Multiple teams may give local anesthetic. One goal of this section is to ensure that the maximum dose of local anesthetic has not already been reached. A shared mental model is created with the Pediatric Regional Anesthesia Time‐Out Checklist “by expanding information both temporally and spatially: it covers items pertaining to the past and present among a group working together on a shared task” [1].

This research shows that the Checklist is helpful and highly rated by its intended users. Furthermore, this Checklist is useful in addressing scenarios drawn from real‐world cases and is designed on the basis of real‐world errors. The setting in which this research was conducted was also realistic in that it involved teams of an attending and trainee working together, similar to how regional anesthesia is often performed.

Worldwide, many different hospital settings exist where pediatric regional anesthesia is practiced. Our study design, including scenarios and survey questions (Supporting Information) also provides a blueprint for testing the Checklist at individual institutions. Based on the responses to these surveys at a local institution, institutional leaders may add or change items on the checklist for local use. The World Health Organization surgical safety checklist initiative supports this practice, stating that for checklists, “additions and modifications to fit local practice are encouraged” [18]. One example of this adaptation might be to confirm that written consent was obtained, for institutions where this is required. We believe that the adaptation process must be performed at every institution that aims to use time‐out checklists successfully. We intend for the results of this study to serve as a model of the iterative design testing process to develop time‐out checklists to support local practice [19].

Our survey revealed that both attendings and trainees thought highly of the role of checklists in regional anesthesia. This may have contributed to our result of a large number of safety items performed after Checklist training. This suggests that in settings where less favorable opinions on checklists exist, more education on the utility of checklists may be needed as part of checklist implementation.

### 4.3 Limitations

This study has limitations. These limitations include the simulated environment, the setting, and the possibility of a learning effect. This research took place in a simulated environment and, therefore, may not reflect subject's real‐world actions. Nevertheless, subjects agreed that the scenarios were realistic and prompted genuine responses. Furthermore, applicability to the real‐world was improved by the fact that scenarios were sourced from available nationally recognized databases and the literature and corresponded with the demographics of the patients in the PRAN database.

This study was performed at a single large children's hospital. Subjects were familiar with working in pairs consisting of a trainee and an attending. These results may not apply to situations in which this pairing is not common. Additionally, the hospital at which these data were collected has a strong safety culture and Quality and Safety program, including regular Morbidity and Mortality conferences. All teams had at least one member with at least 5 years of regional anesthesia experience and who had performed at least five pediatric anesthesia regional anesthetics per month over the past year. Therefore, our conclusions may not apply to settings with a lower clinical volume or different hospital culture. Still, almost half of procedures in the United States take place at children's hospitals, showing the wide applicability of research performed in this setting [20].

A key component of checklist adoption is clinicians' opinions that checklists improve real‐world patient safety. Although this study took place in a simulated environment, all subjects agreed that patients were safe during scenarios using the checklist, whereas some participants were neutral in their perception of patient safety for scenarios that took place prior to checklist introduction.

To test subjects' decision‐making and clinical judgment before and after Checklist training, some scenarios contained programmed errors for which it was expected that the subjects would choose not to perform the regional anesthetic. After checklist training, subjects performed as expected more often. Since checklist training occurred halfway through the performance of scenarios, it is possible that this could represent a learning effect. However, each of the 12 simulation exercises was a different clinical scenario. We also think that a learning effect is less likely because at least one member of the team in every group had extensive recent experience performing pediatric regional anesthetics. Additionally, during the second half of the study, subjects were likely experiencing more fatigue, yet fewer errors occurred, further showing the value of the Checklist for increasing safety.

In this study, subjects were instructed that each scenario ended when the block needle was inserted. This is a limitation, as this instruction could have led to the expectation that the block would always be performed. We think that this possibility is unlikely, however, given that the subjects usually did not perform the block in scenarios with a programmed error. After all 11 attending‐resident dyads participated, 44 scenarios had occurred in which the expectation was not to perform the block. In most (41/44 or 93%) of the 44 scenarios where needle insertion was not expected, the subjects did not perform the block.

The Pediatric Regional Anesthesia Time‐Out Checklist may be particularly valuable for less experienced personnel or teams that do not have extensive experience working together, however, another limitation was that our sample size was not large enough to look at these variables. Another limitation could be that not all of the blocks involved laterality, which was a scored safety item. However, all 11 dyads were given the same scenarios.

## 5 Conclusions

After training on the Pediatric Regional Anesthesia Time‐Out Checklist, the number of safety items performed prior to performing a simulated regional anesthetic increased. This result supports increased adoption of safety checklists prior to pediatric regional anesthesia. Further research should examine the adaptation of this checklist for local needs. Future studies should also examine whether the Pediatric Regional Anesthesia Time‐Out Checklist leads to fewer errors and increased patient safety in real‐world settings.

## Ethics Statement

Approval for the main study was obtained from the University of Colorado School of Medicine Institutional Review Board, #18‐0074.

## Consent

Subjects were recruited from anesthesiology faculty, residents, and fellows at Children's Hospital Colorado with verbal consent for participation and written consent for video recording.

## Conflicts of Interest

The authors declare no conflicts of interest.

## Permission to Reproduce Material From Other Sources

Permission to reproduce the Regional Anesthesia Time‐Out Checklist Obtained from BMJ (form enclosed as Supporting Information).

## Supporting information


Data S1.

## Data Availability

Data is available as Supporting Information for this manuscript.
