# Supplementary material for: Evaluation of the Pediatric Regional Anesthesia Time‐Out Checklist: A Simulation Study
Source: Paediatr Anaesth. 2025 Jan 24;35(6):430–8. doi: 10.1111/pan.15069 (PMC12060083; doi:10.1111/pan.15069)
Supplement: Supplementary file 1 — Data S1. [file PAN-35-430-s001.zip › SupplementalMaterial_All_Scenarios.docx]

Practice Scenario **Anesthesia Resident**:

- WK, a 16 year old boy, 68 kg, penicillin causes rash, MRN 2284106, DOB 2-30-2000, ASA II, presents for an ACL repair after a basketball injury. He also suffers from mild asthma, for which he takes albuterol as needed. The site is marked by the surgeon. The plan is to place a single shot femoral block at the end of the case for post-operative pain. The patient is under general anesthesia, the surgery is over, and it is time to place the block.

Additional Information obtained by Anesthesia Resident:

- You have set the room, including having the equipment available and set up and the LAST treatment kit and intralipid available

**PERFORM SCENARIO**

**PLEASE HAND THIS SHEET BACK TO YOUR PRECEPTOR**

Practice Scenario **Anesthesia Attending**:

- WK, a 16 year old boy, 68 kg, penicillin causes rash, MRN 2284106, DOB 2-30-2000, ASA II, presents for an ACL repair after a basketball injury. He also suffers from mild asthma, for which he takes albuterol as needed. The site is marked by the surgeon. The plan is to place a single shot femoral block at the end of the case for post-operative pain. The patient is under general anesthesia, the surgery is over, and it is time to place the block.

Additional Information obtained by Anesthesia Attending:

- The patient’s mother is very worried about pain after the surgery. She wants to minimize narcotics because she doesn’t want her son to be too sleepy to catch up on his homework over the weekend.

**PERFORM SCENARIO**

**PLEASE HAND THIS SHEET BACK TO YOUR PRECEPTOR**

**SCENARIO 1 ANESTHESIA RESIDENT**: Internal Use: 2

Scenario: AB, a 9 month old boy, 10 kg, allergic to tape which causes redness, MRN 7391229, DOB 1-8-2018, ASA I, presents for repair of a congenital hydrocele. He has been growing normally and has no other past medical history. The urologist requests a caudal at the beginning of the case. The child’s groin is marked by the surgeon. General anesthesia has been induced.

Additional information obtained by **ANESTHESIA RESIDENT**:

The surgeon told you that a single shot caudal is fine, a catheter is not needed.

**PERFORM SCENARIO**

**PLEASE HAND THIS SHEET BACK TO YOUR PRECEPTOR**

**SCENARIO 1 ANESTHESIA ATTENDING**: Internal Use: 2

Scenario: AB, a 9 month old boy, 10 kg, allergic to tape which causes redness, MRN 7391229, DOB 1-8-2018, ASA I, presents for repair of a congenital hydrocele. He has been growing normally and has no other past medical history. The urologist requests a caudal at the beginning of the case. The child’s groin is marked by the surgeon. General anesthesia has been induced.

Additional information obtained by **ANESTHESIA ATTENDING**:

You have reset the room while your resident takes a coffee break. The equipment is available and set up, and your LAST treatment kit is available.

**PERFORM SCENARIO**

**PLEASE HAND THIS SHEET BACK TO YOUR PRECEPTOR**

**PLEASE HAND THIS SHEET BACK TO YOUR PRECEPTOR**

**SCENARIO 2 ANESTHESIA RESIDENT** Internal Use: 1

ME, a 9 year old girl, 42 kg, allergic to penicillin which causes a rash, MRN 7381029, DOB 4-8-2009, ASA II presents for an right ACL repair after an ice skating injury two weeks ago. Besides minor scrapes that are now well healed, there are no other injuries. The child suffers from moderately severe asthma for which she takes daily-inhaled fluticasone, needing albuterol only with upper respiratory infections. She has never been to the hospital or emergency room for asthma, and has never needed oral or IV steroids for her asthma. She is not wheezing today. The orthopedist requests a femoral single shot block after the induction of general anesthesia for post-operative pain. Her right knee is marked by the surgeon. General anesthesia has been induced.

Additional information obtained by **ANESTHESIA RESIDENT**:

Your equipment is available and set up, and your LAST treatment kit is available. The surgeon has told you that she also plans to infiltrate 20 cc of 0.25% bupivacaine intraarticularly at the end of the case.

**PERFORM SCENARIO**

**PLEASE HAND THIS SHEET BACK TO YOUR PRECEPTOR**

**SCENARIO 2 ANESTHESIA ATTENDING**: Internal Use: 1

Scenario ME, a 9 year old girl, 42 kg, allergic to penicillin which causes a rash, MRN 7381029, DOB 4-8-2009, ASA II presents for an right ACL repair after an ice skating injury two weeks ago. Besides minor scrapes that are now well healed, there are no other injuries. The child suffers from moderately severe asthma for which she takes daily-inhaled fluticasone, needing albuterol only with upper respiratory infections. She has never been to the hospital or emergency room for asthma, and has never needed oral or IV steroids for her asthma. She is not wheezing today. The orthopedist requests a femoral single shot block after the induction of general anesthesia for post-operative pain. Her right knee is marked by the surgeon. General anesthesia has been induced.

Additional information obtained by **ANESTHESIA ATTENDING**:

No other information.

**PERFORM SCENARIO**

**PLEASE HAND THIS SHEET BACK TO YOUR PRECEPTOR**

**PLEASE HAND THIS SHEET BACK TO YOUR PRECEPTOR**

**SCENARIO 3 ANESTHESIA RESIDENT**: Internal Use: 9

Scenario: FD, a 16 year old, 61 kg woman, NKDA, MRN 2539584, ASA I, DOB 6-1-2001, underwent a laparoscopic abdominal exploration to evaluate for inflammation and infection of the ovaries. At the end of the case, she wakes up with 10/10 pain, hysterical, unresponsive to medication. The team decides to perform a rectus sheath block in the operating room.

Additional information obtained by **ANESTHESIA RESIDENT**:

Intraoperatively after wakeup: Medication given after wakeup for pain is 3 doses of 0.4 mg hydromorphone

**PERFORM SCENARIO**

**PLEASE HAND THIS SHEET BACK TO YOUR PRECEPTOR**

**SCENARIO 3 ANESTHESIA ATTENDING** Internal Use: 9

Scenario: FD, a 16 year old, 61 kg woman, NKDA, MRN 2539584, ASA I, DOB 6-1-2001, underwent a laparoscopic abdominal exploration to evaluate for inflammation and infection of the ovaries. At the end of the case, she wakes up with 10/10 pain, hysterical, unresponsive to medication. The team decides to perform a rectus sheath block in the operating room.

Additional information obtained by **ANESTHESIA ATTENDING**

While your resident takes care of the patient in the operating room at the end of the case, you make sure the equipment is available and set up and you check that the LAST treatment kit is available.

Intraoperatively after wakeup: Medication given after wakeup for pain is 3 doses of 0.4 mg hydromorphone

**PERFORM SCENARIO**

**PLEASE HAND THIS SHEET BACK TO YOUR PRECEPTOR**

**PLEASE HAND THIS SHEET BACK TO YOUR PRECEPTOR**

**SCENARIO 4 ANESTHESIA RESIDENT**: Internal Use: 3

Scenario: EK, a 5 month old boy, 6 kg, NKDA, MRN 2363916, DOB 4-8-2018, ASA I, presents for bilateral Achilles tendon release. Both feet are marked by the surgeon. The plan is to place a single shot caudal at the beginning of the case for post-operative pain. The patient is under general anesthesia, and it is time to place the block.

Additional information obtained by ANESTHESIA RESIDENT:

No additional info

**PERFORM SCENARIO**

**PLEASE HAND THIS SHEET BACK TO YOUR PRECEPTOR**

**SCENARIO 4 ANESTHESIA ATTENDING**: Internal Use: 3

Scenario: EK, a 5 month old boy, 6 kg, NKDA, MRN 2363916, DOB 4-8-2018, ASA I, presents for bilateral Achilles tendon release. Both feet are marked by the surgeon. The plan is to place a single shot caudal at the beginning of the case for post-operative pain. The patient is under general anesthesia, and it is time to place the block.

Additional information obtained by ANESTHESIA ATTENDING:

While your resident is at a noon lecture, you set the room, including having the equipment is available and set up. You spend some time looking for the LAST treatment kit and intralipid, but you finally find it.

**PERFORM SCENARIO**

**PLEASE HAND THIS SHEET BACK TO YOUR PRECEPTOR**

**PLEASE HAND THIS SHEET BACK TO YOUR PRECEPTOR**

**SCENARIO 5 ANESTHESIA RESIDENT**: Internal Use: 10

Scenario: GZ, a 6 year old boy, 55 kg, allergic to clindamycin which causes hives, MRN 2254165, DOB 8-16-2007, ASA III, presents for excision of a recurrent left fibular osteosarcoma. He is s/p chemotherapy and radiation, most recently last week. The site is marked by the surgeon. The plan is to place an epidural catheter at the beginning of the case. The patient is under general anesthesia, and it is time to place the block.

Additional information obtained by **ANESTHESIA RESIDENT:**

The patient has not had a recent platelet count. The surgeon assures you that the platelet count 2 weeks ago at an outside hospital closer to the patient’s home was fine. You send a CBC but agree to go into the room before the results return. Right now, you get a message on your pager from the lab that the platelet count is 12.

**PERFORM SCENARIO**

**PLEASE HAND THIS SHEET BACK TO YOUR PRECEPTOR**

**SCENARIO 5 ANESTHESIA ATTENDING**: Internal Use: 10

Scenario: GZ, a 6 year old boy, 55 kg, allergic to clindamycin which causes hives, MRN 2254165, DOB 8-16-2007, ASA III, presents for excision of a recurrent left fibular osteosarcoma. He is s/p chemotherapy and radiation, most recently last week. The site is marked by the surgeon. The plan is to place an epidural catheter at the beginning of the case. The patient is under general anesthesia, and it is time to place the block.

Additional information obtained by ANESTHESIA ATTENDING:

You set up the room while your resident is at lunch, and you have confirmed that the equipment is available and set up and the LAST treatment kit is available.

**PERFORM SCENARIO**

**PLEASE HAND THIS SHEET BACK TO YOUR PRECEPTOR**

**PLEASE HAND THIS SHEET BACK TO YOUR PRECEPTOR**

**SCENARIO 6 ANESTHESIA RESIDENT** Internal Use: 4

Scenario: WS, a 3 year old boy, 12 kg, NKDA, MRN 4142519, DOB 1-3-2018, ASA II. presents for surgery for a wide excision of a right thigh melanoma. His last upper respiratory infection was RSV at 2 months of age, for which he was hospitalized overnight, but did not require the ICU or intubation. You decide to perform a lumbar epidural for post-op pain. The patient is under general anesthesia and it is time to place the block.

Additional information obtained by ANESTHESIA RESIDENT

The equipment is available and set up, and the LAST treatment kit is available. Preoperatively, the mother is very concerned about pain relief for her baby after the surgery.

**PERFORM SCENARIO**

**PLEASE HAND THIS SHEET BACK TO YOUR PRECEPTOR**

**SCENARIO 6 ANESTHESIA ATTENDING** Internal Use: 4

WS, a 3 year old boy, 12 kg, NKDA, MRN 4142519, DOB 1-3-2018, ASA II. presents for surgery for a wide excision of a right thigh melanoma. His last upper respiratory infection was RSV at 2 months of age, for which he was hospitalized overnight, but did not require the ICU or intubation. You decide to perform a lumbar epidural for post-op pain. The patient is under general anesthesia and it is time to place the block.

Additional information obtained by **ANESTHESIA ATTENDING**

The site was not marked when you were originally ready to take the child back for surgery, but you find the surgery resident and he marks the child’s left groin area before the child is brought to the OR.

**PERFORM SCENARIO**

**PLEASE HAND THIS SHEET BACK TO YOUR PRECEPTOR**

**SECOND HALF OF STUDY PRACTICE SCENARIO** Internal Use:

**ANESTHESIA RESIDENT**

EK, a 16 year old girl presents for laparoscopic surgery for ovarian torsion. The site is marked by the surgeon. The plan is to place a TAP block at the end of the case for post-operative pain. The patient is under general anesthesia, and it is time to place the block.

Additional information obtained by Anesthesia Resident:

You have set the room, including having the equipment available and set up and the LAST treatment kit and intralipid available. You are ready to perform the Regional Anesthesia Time Out checklist immediately before the block

**PERFORM SCENARIO**

**PLEASE HAND THIS SHEET BACK TO YOUR PRECEPTOR**

**SECOND HALF OF STUDY PRACTICE SCENARIO** Internal Use:

**ANESTHESIA ATTENDING**

EK, a 16 year old girl presents for laparoscopic surgery for ovarian torsion. The site is marked by the surgeon. The plan is to place a TAP block at the end of the case for post-operative pain. The patient is under general anesthesia, and it is time to place the block.

Additional information obtained by Anesthesia Attending:

The patient and her family are very concerned about post-op pain. You are ready to perform the Regional Anesthesia Time Out checklist

immediately before the block

**PERFORM SCENARIO**

**PLEASE HAND THIS SHEET BACK TO YOUR PRECEPTOR**

**PLEASE HAND THIS SHEET BACK TO YOUR PRECEPTOR**

**SCENARIO 7 ANESTHESIA RESIDENT**: Internal Use: 8

Scenario: CL, an 8 year old girl, 30 kg, NKDA, MRN 2343512, DOB 1-18-2003, ASA II, presents for excision of a 3x3 inch nevus, suspicious for melanoma, on her lower belly. The site is marked. Her past medical history includes congenital hypothyroidism, well controlled with daily synthroid. The plan is to place a single shot ileoinguinal block at the end of the case. The patient is under general anesthesia, the surgery is completed as expected and it is time to place the block.

Additional information obtained by **ANESTHESIA RESIDENT:**

Preoperatively, the patient mentions to you that she is scared of waking up during the surgery. You reassure her.

**PERFORM SCENARIO**

**PLEASE HAND THIS SHEET BACK TO YOUR PRECEPTOR**

**SCENARIO 7 ANESTHESIA ATTENDING**: Internal Use: 8

Scenario: CL, an 8 year old girl, 30 kg, NKDA, MRN 2343512, DOB 1-18-2003, ASA II, presents for excision of a 3x3 inch nevus, suspicious for melanoma, on her lower belly. The site is marked. Her past medical history includes congenital hypothyroidism, well controlled with daily synthroid. The plan is to place a single shot ileoinguinal block at the end of the case. The patient is under general anesthesia, the surgery is completed as expected and it is time to place the block.

Additional information obtained by **ANESTHESIA ATTENDING:**

You set the room, including having the equipment is available and set up, while you sent the resident to lunch. The LAST treatment kit is also available.

**PERFORM SCENARIO**

**PLEASE HAND THIS SHEET BACK TO YOUR PRECEPTOR**

**PLEASE HAND THIS SHEET BACK TO YOUR PRECEPTOR**

**SCENARIO 8 ANESTHESIA RESIDENT**: Internal Use: 5

Scenario: NW, a 7 month old boy, 8 kg, NKDA, MRN 1822986, DOB 3-21-2018, ASA III, presents for repair of a bilateral inguinal hernia. He was born at 29 weeks gestation, and is currently on 0.5 L oxygen in the step-down unit. The site is marked by the surgeon. The plan is to place a caudal block at the beginning of the case. The patient is under general anesthesia, and it is time to place the block.

Additional information obtained by **ANESTHESIA RESIDENT:**

The surgery resident asks you for 25 mL of 1% lidocaine with epinephrine from your cart for local infiltration. You question both his dose of local and the need for any additional local because the patient will also be given a caudal block, and he agrees that additional local will not be needed.

**PERFORM SCENARIO**

**PLEASE HAND THIS SHEET BACK TO YOUR PRECEPTOR**

**SCENARIO 8 ANESTHESIA ATTENDING**: Internal Use: 5

Scenario: NW, a 7 month old boy, 8 kg, NKDA, MRN 1822986, DOB 3-21-2018, ASA III, presents for repair of a bilateral inguinal hernia. He was born at 29 weeks gestation, and is currently on 0.5 L oxygen in the step-down unit. The site is marked by the surgeon. The plan is to place a caudal block at the beginning of the case. The patient is under general anesthesia, and it is time to place the block.

Additional information obtained by **ANESTHESIA ATTENDING:**

You set up the case while you give your resident a break, and before the case, you make sure that the equipment is available and set up and that the LAST treatment kit is available.

**PERFORM SCENARIO**

**PLEASE HAND THIS SHEET BACK TO YOUR PRECEPTOR**

**PLEASE HAND THIS SHEET BACK TO YOUR PRECEPTOR**

**SCENARIO 9 ANESTHESIA RESIDENT**: Internal Use: 12

Scenario: LM, an 18 year old boy, 158 kg, NKDA, MRN 31226399, DOB 4-3-2000, ASA II. He presents for extensive foot debridement for an infected wound with cellulitus after hurting his foot playing soccer 4 weeks ago, but not telling his parents that his foot hurt or seeking medical care until 2 days ago. He also has type II diabetes, for which he takes metformin daily. The patient had 3 days of intractable nausea and vomiting after his last surgery, so in conjunction with the surgeon, the anesthesiologist that was consulted yesterday for an in-house pre-op came up with the plan that a lumbar epidural catheter would be placed under general anesthesia at the start of the case for post-operative pain. The surgical site is marked. The patient is under general anesthesia and it is time to place the block.

Additional information obtained by **ANESTHESIA RESIDENT:**

The equipment is available and set up, and the LAST treatment kit is available. The patient has been on enoxaparin twice daily in house for the last 2 days to avoid DVTs due to his immobility and obesity, receiving his last dose 4 hours ago.

**PERFORM SCENARIO**

**PLEASE HAND THIS SHEET BACK TO YOUR PRECEPTOR**

**SCENARIO 9 ANESTHESIA ATTENDING**: Internal Use: 12

Scenario: LM, an 18 year old boy, 158 kg, NKDA, MRN 31226399, DOB 4-3-2000, ASA II. He presents for extensive foot debridement for an infected wound with cellulitus after hurting his foot playing soccer 4 weeks ago, but not telling his parents that his foot hurt or seeking medical care until 2 days ago. He also has type II diabetes, for which he takes metformin daily. The patient had 3 days of intractable nausea and vomiting after his last surgery, so in conjunction with the surgeon, the anesthesiologist that was consulted yesterday for an in-house pre-op came up with the plan that a lumbar epidural catheter would be placed under general anesthesia at the start of the case for post-operative pain. The surgical site is marked. The patient is under general anesthesia and it is time to place the block.

Additional information obtained by **ANESTHESIA ATTENDING:**

No additional info

**PERFORM SCENARIO**

**PLEASE HAND THIS SHEET BACK TO YOUR PRECEPTOR**

**PLEASE HAND THIS SHEET BACK TO YOUR PRECEPTOR**

**SCENARIO 10 ANESTHESIA RESIDENT**: Internal Use: 7

Scenario: WE, a 16 year old girl, 50 kg, NKDA, MRN 1221315, DOB 3-11-2002, ASA I, presents for repair of an ankle fracture of the lateral malleolus. She is otherwise healthy. The site is marked by the surgeon. The plan is to place a single shot sciatic block at the beginning of the case for post-op pain. The patient is under general anesthesia, and it is time to place the block.

Additional information obtained by **ANESTHESIA RESIDENT**:

You have confirmed that the equipment is available and set up and the LAST treatment kit is available.

**PERFORM SCENARIO**

**AFTER SCENARIO**

Please privately (no sharing!) answer the question:

Would you perform (or should the team perform) a regional block in this patient? YES NO (circle one).

Are there any issues with doing so? If so, what are t

**PLEASE HAND THIS SHEET BACK TO YOUR PRECEPTOR**

**SCENARIO 10 ANESTHESIA ATTENDING**: Internal Use: 7

Scenario: WE, a 16 year old girl, 50 kg, NKDA, MRN 1221315, DOB 3-11-2002, ASA I, presents for repair of an ankle fracture of the lateral malleolus. She is otherwise healthy. The site is marked by the surgeon. The plan is to place a single shot sciatic block at the beginning of the case for post-op pain. The patient is under general anesthesia, and it is time to place the block.

Additional information obtained by **ANESTHESIA ATTENDING**:

No further information

**PERFORM SCENARIO**

**AFTER SCENARIO**

Please privately (no sharing!) answer the question:

Would you perform (or should the team perform) a regional block in this patient? YES NO (circle one).

Are there any issues with doing so? If so, what are they?

**PLEASE HAND THIS SHEET BACK TO YOUR PRECEPTOR**

**PLEASE HAND THIS SHEET BACK TO YOUR PRECEPTOR**

**SCENARIO 11 ANESTHESIA RESIDENT**: Internal Use: 11

Scenario: NH, a 1 year old boy, 11 kg, NKDA, MRN 1832326, DOB 12-11-2017, ASA I, presents for repair of an undescended testicle. He is otherwise healthy. The site is marked by the surgeon. The plan is to place a caudal block at the beginning of the case. The patient is under general anesthesia, and it is time to place the block.

Additional information obtained by Anesthesia Resident:

           Before the case, the mom asks you ‘do you think this surgery is really needed? My baby is healthy.’ You call the surgeon to speak with the mom again before the procedure, and she is reassured and consents to the surgery.

**PERFORM SCENARIO**

**PLEASE HAND THIS SHEET BACK TO YOUR PRECEPTOR**

**SCENARIO 11 Anesthesia Attending**: Internal Use: 11

Scenario: NH, a 1 year old boy, 11 kg, NKDA, MRN 1832326, DOB 12-11-2017, ASA I, presents for repair of an undescended testicle. He is otherwise healthy. The site is marked by the surgeon. The plan is to place a caudal block at the beginning of the case. The patient is under general anesthesia, and it is time to place the block.

Additional information obtained by ANESTHESIA ATTENDING:

     In between cases, you sent your resident to follow up on a patient from earlier that day, and you set the room and made sure that the equipment is available and set up and the LAST treatment kit available. The resident has returned.

**PERFORM SCENARIO**

**PLEASE HAND THIS SHEET BACK TO YOUR PRECEPTOR**

**SCENARIO 12 ANESTHESIA RESIDENT** Internal Use: 6

Scenario: LM, a 6 month old boy, 8 kg, NKDA, MRN 2934105, DOB 2-16-2018, ASA I, presents for a circumcision for phymosis. He is otherwise healthy. The site is marked by the surgeon. The plan is to place a single shot caudal block at the beginning of the case. The patient is under general anesthesia, and it is time to place the block.

Additional information obtained by ANESTHESIA RESIDENT: You have confirmed that the equipment is available and set up and the LAST treatment kit is available.

**PERFORM SCENARIO**

**AFTER SCENARIO**

Please privately (no sharing!) answer the question:

Would you perform (or should the team perform) a regional block in this patient? YES NO (circle one).

Are there any issues with doing so? If so, what are they?

**PLEASE HAND THIS SHEET BACK TO YOUR PRECEPTOR**

**SCENARIO 12 ANESTHESIA ATTENDING**: Internal Use: 6

Scenario: LM, a 6 month old boy, 8 kg, NKDA, MRN 2934105, DOB 2-16-2018, ASA I, presents for a circumcision for phymosis. He is otherwise healthy. The site is marked by the surgeon. The plan is to place a single shot caudal block at the beginning of the case. The patient is under general anesthesia, and it is time to place the block.

Additional information obtained by **ANESTHESIA ATTENDING:**

The surgeon had originally planned for a penile block. You ask him if it would be OK to perform a caudal block instead for teaching purposes. The surgeon agrees, and the parents also agree and consent to this.

**PERFORM SCENARIO**
