# Supplementary material for: Evaluation of the Pediatric Regional Anesthesia Time‐Out Checklist: A Simulation Study
Source: Paediatr Anaesth. 2025 Jan 24;35(6):430–8. doi: 10.1111/pan.15069 (PMC12060083; doi:10.1111/pan.15069)
Supplement: Supplementary file 1 — Data S1. [file PAN-35-430-s001.zip › SupplementalMaterial_ScenariosWithProgrammedErrors.docx]

**SCENARIO A**

Scenario: FD, a 16 year old, 61 kg woman, NKDA, MRN 2539584, ASA I, DOB 6-1-2001, underwent a laparoscopic abdominal exploration to evaluate for inflammation and infection of the ovaries. At the end of the case, she wakes up with 10/10 pain, hysterical, unresponsive to medication. The team decides to perform a rectus sheath block in the operating room.

Additional information obtained by **ANESTHESIA RESIDENT**:

Intraoperatively after wakeup: Medication given after wakeup for pain is 3 doses of 0.4 mg hydromorphone

Additional information obtained by **ANESTHESIA ATTENDING**

While your resident takes care of the patient in the operating room at the end of the case, you make sure the equipment is available and set up and you check that the LAST treatment kit is available.

Intraoperatively after wakeup: Medication given after wakeup for pain is 3 doses of 0.4 mg hydromorphone

At the end of the surgery, before placing the dressing, the surgeon infiltrated 20 mL ropivacaine 0.75% into the intraperitoneal cavity

**SCENARIO B**

Scenario: GZ, a 6 year old boy, 55 kg, allergic to clindamycin which causes hives, MRN 2254165, DOB 8-16-2007, ASA III, presents for excision of a recurrent left fibular osteosarcoma. He is s/p chemotherapy and radiation, most recently last week. The site is marked by the surgeon. The plan is to place an epidural catheter at the beginning of the case. The patient is under general anesthesia, and it is time to place the block.

Additional information obtained by **ANESTHESIA RESIDENT:**

The patient has not had a recent platelet count. The surgeon assures you that the platelet count 2 weeks ago at an outside hospital closer to the patient’s home was fine. You send a CBC but agree to go into the room before the results return. Right now, you get a message on your pager from the lab that the platelet count is 12.

Additional information obtained by **ANESTHESIA ATTENDING**:

No further information

**SCENARIO C**

Scenario: NH, a 1 year old boy, 11 kg, NKDA, MRN 1832326, DOB 12-11-2017, ASA I, presents for repair of an undescended testicle. He is otherwise healthy. The site is marked by the surgeon. The plan is to place a caudal block at the beginning of the case. The patient is under general anesthesia, and it is time to place the block.

Additional information obtained by Anesthesia Resident:

           Before the case, the mom asks you ‘do you think this surgery is really needed? My baby is healthy.’ You call the surgeon to speak with the mom again before the procedure, and she is reassured and consents to the surgery.

The surgical team has just infiltrated 9 cc of 0.25% bupivacaine for a penile block.

Additional information obtained by ANESTHESIA ATTENDING:

     In between cases, you sent your resident to follow up on a patient from earlier that day, and you set the room and made sure that the equipment is available and set up and the LAST treatment kit available. The resident has returned.

**SCENARIO D**

Scenario: LM, an 18 year old boy, 158 kg, NKDA, MRN 31226399, DOB 4-3-2000, ASA II. He presents for extensive foot debridement for an infected wound with cellulitus after hurting his foot playing soccer 4 weeks ago, but not telling his parents that his foot hurt or seeking medical care until 2 days ago. He also has type II diabetes, for which he takes metformin daily. The patient had 3 days of intractable nausea and vomiting after his last surgery, so in conjunction with the surgeon, the anesthesiologist that was consulted yesterday for an in-house pre-op came up with the plan that a lumbar epidural catheter would be placed under general anesthesia at the start of the case for post-operative pain. The surgical site is marked. The patient is under general anesthesia and it is time to place the block.

Additional information obtained by **ANESTHESIA RESIDENT:**

The equipment is available and set up, and the LAST treatment kit is available. The patient has been on enoxaparin twice daily in house for the last 2 days to avoid DVTs due to his immobility and obesity, receiving his last dose 4 hours ago.

Additional information obtained by **ANESTHESIA ATTENDING:**

No additional info
