# Supplementary material for: Evaluation of the Pediatric Regional Anesthesia Time‐Out Checklist: A Simulation Study
Source: Paediatr Anaesth. 2025 Jan 24;35(6):430–8. doi: 10.1111/pan.15069 (PMC12060083; doi:10.1111/pan.15069)
Supplement: Supplementary file 1 — Data S1. [file PAN-35-430-s001.zip › SupplementalMaterial_SurveyResults.docx]

**Supplemental Material: Survey Results**

1. **Table: Demographics Attendings**

| Group | Number of Years as An Attending | Board Certified | Fellowship Trained | Additional Residency Besides Anesthesiology | Gender | Age | Number of Regional Blocks over past yr per month average in patients less than age 21 administered or supervised | Number of Years I have been performing regional anesthetics |
| --- | --- | --- | --- | --- | --- | --- | --- | --- |
| 1 | 3 | Y | Y | Y | M | 40 | >10 | 5-10 |
| 2 | 14 | Y | Y | none | M | 43 | 5-10 | >10 |
| 3 | 9 | Y | Y | No | M | 41 | >10 | >10 |
| 4 | 10 | Y | Y. Peds anesthesia | No | F | 50 | 2-5 | >10 |
| 5 | 11 | Y | Y | No | M | 50 | >10 | >10 |
| 6 | 10 | Y | Y | No | M | 45 | 2-5 | >10 |
| 7 | 3 | Y | Y | Regional Fellowship | F | 33 | >10 | 5-10 |
| 8 | 7 | Y | Y | No | M | 42 | >10 | 5-10 |
| 9 | 6 | Y | Y |  | M | 40 | 5-10 | 5-10 |
| 10 | 40 | Anesthesia, pediatrics, pediatric critical care | Anesthesia, pediatrics, pediatric critical care | pediatrics | M | 67 | >10 | >10 |
| 11 | 1 | Y | Y | no | F | 32 | 5-10 | 2-5 |
| Mean(SD) | 10.4(10.5) |  |  |  |  | 43.9(9.6) |  |  |

1. **Table: Background First Half of Study Survey Attending**

**Likert scale: 1 = None; 5 = Great Deal**

| Group | **How many times worked with other subject in this group (fellow/resident) previously** | How much **training** have you previously had regarding the use of checklists as a part of your **normal** practice (e.g., the time-out checklist or WHO surgical safety checklist)? | How much **experience** do you have using checklists as a part of your **normal** practice (e.g., the time-out checklist or WHO surgical safety checklist)? |
| --- | --- | --- | --- |
| 1 | 3 | 4 | 5 |
| 2 | 5 | 4 | 4 |
| 3 | 0 | 4 | 4 |
| 4 | 2 | 3 | 5 |
| 5 | 4 | 4 | 4 |
| 6 | 10 | 5 | 5 |
| 7 | 0 | 3 | 4 |
| 8 | 10 | 5 | 5 |
| 9 | 5 | 4 | 2 |
| 10 | 5 | 5 | 5 |
| 11 | 1 | 3 | 3 |
| Median [IQR] |  | 4[3,5] | 4[4,5] |

1. **Table: First Half of Study Opinions Attending**

| Group | Overall, during the last 6 regional anesthesia scenarios, how safe did I feel that the patients were?  1 = Very Unsafe 5 = Very Safe | Why? | Overall, during the last 6 regional anesthesia scenarios, how good was the teamwork?  1 = Very Poor 5 = Very Good | Why? |
| --- | --- | --- | --- | --- |
| 1 | 5 | We thought of many scenarios to increase safety, including contraindications to neuraxial | 5 | We had eye contact, confirmed + agreed on points of concern |
| 2 | 5 | [blank] | 4 | [blank] |
| 3 | 3 | [blank] | 4 | [blank] |
| 4 | 4 | Penile block for testicular surgery was concerning | 5 | [blank] |
| 5 | 4 | [blank] | 5 | [blank] |
| 6 | 5 | [blank] | 5 | [blank] |
| 7 | 5 | [blank] | 5 | [blank] |
| 8 | 5 | [blank] | 5 | [blank] |
| 9 | 5 | [blank] | 5 | [blank] |
| 10 | 5 | [blank] | 5 | [blank] |
| 11 | 5 | [blank] | 4 | [blank] |
| Median[IQR] |  |  |  |  |

1. **Table: Demographics Resident/Fellow**

| Group | Current Position | Board Certified | Fellowship Trained | Additional Residency Besides Anesthesiology | Gender | Age | Number of Regional Blocks per month average in patients less than age 21 | Number of Years I have been performing regional anesthetics |
| --- | --- | --- | --- | --- | --- | --- | --- | --- |
| 1 | Fellow |  | Peds Anesthesia | [blank] | M | 34 | >10 | 2-5 |
| 2 | Fellow |  | Peds Anesthesia | none | M | 31 | 5-10 | 2-5 |
| 3 | CA-2 |  |  |  | F | 31 | 2-5 | 1-2 |
| 4 | Fellow |  | Pediatric Anesthesiology |  | F | 32 | 2-5 | 1-2 |
| 5 | Fellow |  | Peds Anesthesia | Pediatrics | F | 35 | 2-5 | 2-5 |
| 6 | Fellow |  | Peds |  | F | 30 | 5-10 | 2-5 |
| 7 | CA-3 |  | No | No | M | 32 | 0 | None |
| 8 | Fellow |  | Peds anesthesia | No | M | 31 | >10 | 2-5 |
| 9 | Fellow |  | Peds | n/a | M | 33 | >10 | 2-5 |
| 10 | Fellow |  | Peds anesthesia | No | M | 31 | >10 | 2-5 |
| 11 | CA-2 |  |  |  | M | 29 | 5-10 | 2-5 |
| Total |  |  |  |  |  | 31.7(1.7) mean(SD) |  |  |

1. **Table: Background First Half of Study Survey Resident/Fellow**

**Likert scale: 1 = None; 5 = Great Deal**

| Group | **How many times worked with other subject in this group (attending) previously** | How much **training** have you previously had regarding the use of checklists as a part of your **normal** practice (e.g., the time-out checklist or WHO surgical safety checklist)? | How much **experience** do you have using checklists as a part of your **normal** practice (e.g., the time-out checklist or WHO surgical safety checklist)? |
| --- | --- | --- | --- |
| 1 | 3 | 4 | 5 |
| 2 | 10 | 3 | 5 |
| 3 | 0 | 2 | 2 |
| 4 | 2 | 4 | 4 |
| 5 | 4 | 5 | 5 |
| 6 | 5 | 5 | 4 |
| 7 | 0 | 4 | 4 |
| 8 | 10 | 5 | 5 |
| 9 | 5 | 5 | 5 |
| 10 | 5 | 4 | 4 |
| 11 | 1 | 5 | 5 |
| Median[IQR] |  | 4[4,5] | 5[4,5] |

1. **Table: First Half of Study Opinions Resident/Fellow**

| Group | Overall, during the last 6 regional anesthesia scenarios, how safe did I feel that the patients were?  1 = Very Unsafe 5 = Very Safe | Why? | Overall, during the last 6 regional anesthesia scenarios, how good was the teamwork?  1 = Very Poor 5 = Very Good | Why? |
| --- | --- | --- | --- | --- |
| 1 | 4 | There were a couple scenarios where we thought a block was not indicated and had no issue not proceeding with it with the patient’s interests at heart | [did not answer] | [did not answer] |
| 2 | 3 | -Surgeons performing incorrect block  -Patient receiving lovenox + told they would be getting an epidural | 5 | Recognizing that different information provided to both attending and fellow – important to close loop |
| 3 | 3 | Miscommunication with surgery team re: woke-up, labs, plan, what they have already injected | 5 | Great between my attending and me |
| 4 | 5 | [blank] | 5 | [blank] |
| 5 | 4 | There was missed important info about some pts with incomplete info, but I think good communication between us caught the important info + kept patients safe | 5 | [blank] |
| 6 | 4 | [blank] | 5 | [blank] |
| 7 | 5 | There was excellent communication with my attending | 5 | Strong communication |
| 8 | 5 | [blank] | 5 | [blank] |
| 9 | 5 | [blank] | 5 | [blank] |
| 10 | 5 | [blank] | 5 | [blank] |
| 11 | 5 | ? safety first, then teamwork | 5 | Great communication and imagination |

1. **Table: After Study Opinions Attending Part 1**

| Group | Overall, during the last 6 regional anesthesia scenarios, how safe did I feel that the patients were? 1 = Very Unsafe 5 = Very Safe | Why? | Overall, during the last 6 regional anesthesia scenarios, how good was the teamwork?  1 = Very Poor 5 = Very Good | Why? |
| --- | --- | --- | --- | --- |
| 1 | 5 | [blank] | 5 | [blank] |
| 2 | 5 | [blank] | 5 | [blank] |
| 3 | 4 | [blank] | 4 | [blank] |
| 4 | 5 | Stopped to resolve issues we had before proceeding | 5 | [blank] |
| 5 | 5 | [blank] | 5 | [blank] |
| 6 | 5 | Checklists! | 5 | Checklists forged team participation |
| 7 | 5 | [blank] | 5 | [blank] |
| 8 | 5 | [blank] | 5 | [blank] |
| 9 | 5 | [blank] | 5 | [blank] |
| 10 | 5 | [blank] | 5 | [blank] |
| 11 | 5 | Checklist was helpful to catch any things we might have missed | 5 | [blank] |

1. **Table: After Study Opinions Resident Part 1**

| Group | Overall, during the last 6 regional anesthesia scenarios, how safe did I feel the patients were? 1 = Very Unsafe 5 = Very Safe | Why? | Overall, during the last 6 regional anesthesia scenarios, how good was the teamwork?  1 = Very Poor 5 = Very Good | Why? |
| --- | --- | --- | --- | --- |
| 1 | 5 | [blank] | 5 | [blank] |
| 2 | 5 | Good communication between attending and fellow | 5 | Good communication between attending and fellow |
| 3 | 5 | Clearly understood plan/previous intervention | 5 | [blank] |
| 4 | 5 | Went through pertinent safety info on checklist | 5 | Clear communication |
| 5 | 5 | [blank] | 5 | [blank] |
| 6 | 5 | [blank] | 5 | [blank] |
| 7 | 5 | Awesome checklist | 5 | Communication |
| 8 | 5 | [blank] | 5 | [blank] |
| 9 | 5 | [blank] | 5 | [blank] |
| 10 | 5 | [blank] | 5 | [blank] |
| 11 | 5 | Checklist makes things safer | 5 | Good communication |

1. **Table: After Study Opinions Attending Part 2 Checklist Design and Functionality**

**1 = Strongly Disagree 2 = Disagree 3 = Neutral 4 = Agree 5 = Strongly Agree**

|  | Group | 1. It was easy to read the checklist | 1. Overall, the checklist is too long. |
| --- | --- | --- | --- |
| Attending | 1 | 5 | 1 |
|  | 2 | 4 | 2 |
|  | 3 | 4 | 2 |
|  | 4 | 5 | 3 |
|  | 5 | 5 | 2 |
|  | 6 | 5 | 3 |
|  | 7 | 5 | 2 |
|  | 8 | 5 | 2 |
|  | 9 | 5 | 3 |
|  | 10 | 5 | 3 |
|  | 11 | 5 | 2 |
|  |  |  |  |
| Trainee | 1 | 4 | 3 |
|  | 2 | 5 | 2 |
|  | 3 | 5 | 2 |
|  | 4 | 5 | 4 |
|  | 5 | 5 | 2 |
|  | 6 | 5 | 4 |
|  | 7 | 5 | 2 |
|  | 8 | 4 | 3 |
|  | 9 | 5 | 2 |
|  | 10 | 5 | 1 |
|  | 11 | 5 | 2 |
|  |  |  |  |

1. **Table: After Study Opinions Attending Part 3 Checklist Design and Functionality**

**1 = Strongly Disagree 2 = Disagree 3 = Neutral 4 = Agree 5 = Strongly Agree**

| Group | 1. I clearly understood what I was supposed to do/think about relative to items in the PREOPERATIVELY section of the checklist. | 1. The PREOPERATIVELY section contains all the steps that arenormal essential. | What steps do you think should be added? |
| --- | --- | --- | --- |
| 1 | 5 | 5 | [blank] |
| 2 | 4 | 4 | Imaging studies |
| 3 | 4 | 3 | [blank] |
| 4 | 3 | [blank] | “any questions/concerns before starting” |
| 5 | 4 | 5 | [blank] |
| 6 | 4 | 4 | [blank] |
| 7 | 4 | 4 | [blank] |
| 8 | 5 | 5 | [blank] |
| 9 | 5 | 4 | [blank] |
| 10 | 5 | 5 | [blank] |
| 11 | 5 | 5 | [blank] |

1. **Table: After Study Opinions Resident Part 3 Checklist Design and Functionality**

**1 = Strongly Disagree 2 = Disagree 3 = Neutral 4 = Agree 5 = Strongly Agree**

| Group | 1. I clearly understood what I was supposed to do/think about relative to items in the PREOPERATIVELY section of the checklist. | What could be done to improve the usability of this portion of the checklist? | 1. The PREOPERATIVELY section contains all the steps that are essential. | What steps do you think should be added? |
| --- | --- | --- | --- | --- |
| 1 | 5 | [blank] | 4 | [blank] |
| 2 | 4 | N/A | 4 | -Parental concerns about block  -Other regional blocks |
| 3 | 5 | [blank] | 5 | [blank] |
| 4 | 5 | [blank] | 5 | Confirm adequate ventilation and oxygenation and vital signs checked |
| 5 | 3 | [blank] | 3 | [blank] |
| 6 | 5 | [blank] | 5 | [blank] |
| 7 | 3 | [blank] | 4 | [blank] |
| 8 | 5 | [blank] | 5 | [blank] |
| 9 | 5 | Seemed like PT 10 should be in preop section | 4 | Seemed like PT 10 should be in preop section |
| 10 | 5 | [blank] | 4 | Labs not mentioned |
| 11 | 5 | “is this the most appropriate block?” | 4 | “is this the most appropriate block?” |

1. **Table: After Study Opinions Attending Part 4 Checklist Design and Functionality**

**1 = Strongly Disagree 2 = Disagree 3 = Neutral 4 = Agree 5 = Strongly Agree**

| Group | 1. I clearly understood what I was supposed to do relative to items in the IMMEDIATELY BEFORE PROCEDURE section of the checklist. | 1. What could be done to improve the usability of this portion of the checklist? | **H.** The IMMEDIATELY BEFORE PROCEDURE section contains all the steps that are essential. | What steps do you think should be added? |
| --- | --- | --- | --- | --- |
| 1 | 5 | [blank] | 5 | [blank] |
| 2 | 4 |  | 4 | [blank] |
| 3 | 3 | [blank] | 4 | [blank] |
| 4 | 4 | If LAST occurs, where is cart (if not in OR) and who will get it? | 4 | See previous |
| 5 | 4 | [blank] | 5 | [blank] |
| 6 | 5 | [blank] | 4 | [blank] |
| 7 | 4 | [blank] | 4 | [blank] |
| 8 | 5 | [blank] | 5 | [blank] |
| 9 | 5 | [blank] | 4 | [blank] |
| 10 | 4 | [blank] | Not answered | [blank] |
| 11 | 5 | [blank] | 5 | [blank] |

1. **Table: After Study Opinions Resident Part 4 Checklist Design and Functionality**

**1 = Strongly Disagree 2 = Disagree 3 = Neutral 4 = Agree 5 = Strongly Agree**

| Group | 1. I clearly understood what I was supposed to do relative to items in the IMMEDIATELY BEFORE PROCEDURE section of the checklist. | What could be done to improve the usability of this portion of the checklist? | **N.** The IMMEDIATELY BEFORE PROCEDURE section contains all the steps that are essential. | What steps do you think should be added? |
| --- | --- | --- | --- | --- |
| 1 | 4 | [blank] | 4 | [blank] |
| 2 | 2 | [blank] | 3 | Thrombocytopenia, INR in susceptible patients |
| 3 | 5 | [blank] | 5 | [blank] |
| 4 | 5 | [blank] | 5 | [blank] |
| 5 | 3 | [blank] | 4 | [blank] |
| 6 | 5 | [blank] | 4 | [blank] |
| 7 | 5 | [blank] | 4 | [blank] |
| 8 | 5 | [blank] | 5 | [blank] |
| 9 | 5 | [blank] | 5 | [blank] |
| 10 | 5 | None | 4 | [blank] |
| 11 | 2 | Change the wording | 5 | [blank] |

1. **Table: After Study Opinions Attending Part 5 Checklist Design and Functionality**

**1 = Strongly Disagree 2 = Disagree 3 = Neutral 4 = Agree 5 = Strongly Agree**

| Group | **I.** I thought that the items were in the correct order | How would you change the order of the items? |
| --- | --- | --- |
| 1 | 5 | Just add name |
| 2 | 4 | [blank] |
| 3 | 4 | [blank] |
| 4 | 4 | [blank] |
| 5 | 4 | [blank] |
| 6 | 4 | [blank] |
| 7 | 3 | [blank] |
| 8 | 5 | [blank] |
| 9 | 4 | [blank] |
| 10 | 4 | [blank] |
| 11 | 4 | [blank] |

1. **Table: After Study Opinions Resident Part 5 Checklist Design and Functionality**

**1 = Strongly Disagree 2 = Disagree 3 = Neutral 4 = Agree 5 = Strongly Agree**

| Group | **I.** I thought that the items were in the correct order | How would you change the order of the items? |
| --- | --- | --- |
| 1 | 2 | Add block/laterality to done before surgery |
| 2 | 5 | [blank] |
| 3 | 5 | [blank] |
| 4 | 5 | [blank] |
| 5 | 4 | [blank] |
| 6 | 5 | [blank] |
| 7 | 3 | [blank] |
| 8 | 5 | [blank] |
| 9 | 3 | I would prefer PT to be first |
| 10 | 4 | [blank] |
| 11 | 5 | [blank] |

1. **Table: After Study Opinions Attending Part 6 Checklist Design and Functionality**

**1 = Strongly Disagree 2 = Disagree 3 = Neutral 4 = Agree 5 = Strongly Agree**

| Group | The PREOPERATIVELY section takes too long to complete. | The IMMEDIATELY BEFORE PROCEDURE section takes too long to complete. |
| --- | --- | --- |
| 1 | 2 | 2 |
| 2 | 2 | 2 |
| 3 | 1 | 2 |
| 4 | 2 | 2 |
| 5 | 2 | 2 |
| 6 | 3 | 3 |
| 7 | 3 | 4 |
| 8 | 1 | 1 |
| 9 | 2 | 3 |
| 10 | 2 | 2 |
| 11 | 2 | 2 |

1. **Table: After Study Opinions Resident Part 6 Checklist Design and Functionality**

**1 = Strongly Disagree 2 = Disagree 3 = Neutral 4 = Agree 5 = Strongly Agree**

| Group | The PREOPERATIVELY section takes too long to complete. | The IMMEDIATELY BEFORE PROCEDURE section takes too long to complete. |
| --- | --- | --- |
| 1 | 2 | 2 |
| 2 | 2 | 2 |
| 3 | 2 | 2 |
| 4 | 2 | 2 |
| 5 | 2 | 2 |
| 6 | 3 | 3 |
| 7 | 2 | 2 |
| 8 | 2 | 2 |
| 9 | 1 | 1 |
| 10 | 2 | 1 |
| 11 | 2 | 2 |

1. **Table: After Study Opinions Attending Part 8 Scenarios and Checklist Utility A**

**1 = Strongly Disagree 2 = Disagree 3 = Neutral 4 = Agree 5 = Strongly Agree**

| Group | The scenarios were realistic. | The scenarios prompted realistic responses from me. | The checklist will help me provide safer patient care. | I think the Pediatric Regional Anesthesia Timeout Checklist is good for:  Promoting positive team interactions | I think the Pediatric Regional Anesthesia Timeout Checklist is good for:  Establishing a shared “mental model” of the patient and his/her situation among team members |
| --- | --- | --- | --- | --- | --- |
| 1 | 5 | 5 | 5 | 5 | 5 |
| 2 | 4 | 4 | 4 | 4 | 4 |
| 3 | 4 | 4 | 4 | 4 | 4 |
| 4 | 4 | 4 | 4 | 4 | 4 |
| 5 | 4 | 4 | 5 | 4 | 4 |
| 6 | 5 | 5 | 5 | 5 | 5 |
| 7 | 4 | 4 | 4 | 5 | 5 |
| 8 | 4 | 4 | 4 | 5 | 5 |
| 9 | 4 | 4 | 4 | 4 | 4 |
| 10 | 4 | 4 | 4 | 4 | 4 |
| 11 | 4 | 4 | 4 | 4 | 4 |

1. **Table: After Study Opinions Resident Part 8 Scenarios and Checklist Utility A**

**1 = Strongly Disagree 2 = Disagree 3 = Neutral 4 = Agree 5 = Strongly Agree**

| Group | The scenarios were realistic. | The scenarios prompted realistic responses from me. | The checklist will help me provide safer patient care. | I think the Pediatric Regional Anesthesia Timeout Checklist is good for:  Promoting positive team interactions | I think the Pediatric Regional Anesthesia Timeout Checklist is good for:  Establishing a shared “mental model” of the patient and his/her situation among team members |
| --- | --- | --- | --- | --- | --- |
| 1 | 4 | 3 | 4 | 4 | 4 |
| 2 | 5 | 5 | 4 | 5 | 5 |
| 3 | 5 | 5 | 5 | 5 | 5 |
| 4 | 5 | 5 | 5 | 5 | 5 |
| 5 | 5 | 4 | 4 | 4 | 4 |
| 6 | 5 | 5 | 5 | 4 | 4 |
| 7 | 4 | 4 | 4 | 3 | 4 |
| 8 | 4 | 4 | 4 | 4 | 4 |
| 9 | 4 | 4 | 5 | 4 | 4 |
| 10 | 4 | 4 | 4 | 4 | 4 |
| 11 | 5 | 5 | 5 | 5 | 5 |

1. **Table: After Study Opinions Attending Part 8 Scenarios and Checklist Utility B**

**1 = Strongly Disagree 2 = Disagree 3 = Neutral 4 = Agree 5 = Strongly Agree**

| **I think the Pediatric Regional Anesthesia Timeout Checklist is good for:**  **Helping to catch/identify potential issues or confusion associated with:** | | | | | |
| --- | --- | --- | --- | --- | --- |
| Group | A patient’s allergies | A patient’s coagulation ability | A patient’s bleeding tendency | Administering the wrong amount of regional anesthetic given a patient’s weight | The type of surgery or procedure to be performed |
| 1 | 5 | 5 | 5 | 5 | 5 |
| 2 | 4 | 4 | 4 | 4 | 4 |
| 3 | 4 | 4 | 4 | 4 | 4 |
| 4 | 3 | 4 | 4 | 4 | 4 |
| 5 | 5 | 5 | 5 | 5 | 5 |
| 6 | 5 | 5 | 3 | 5 | 5 |
| 7 | 5 | 5 | 5 | 5 | 5 |
| 8 | 5 | 5 | 5 | 5 | 5 |
| 9 | 5 | 5 | 5 | 5 | 5 |
| 10 | 4 | 4 | 4 | 3 | 4 |
| 11 | 3 | 5 | 5 | 2 | 4 |

1. **Table: After Study Opinions Resident Part 8 Scenarios and Checklist Utility B**

**1 = Strongly Disagree 2 = Disagree 3 = Neutral 4 = Agree 5 = Strongly Agree**

| **I think the Pediatric Regional Anesthesia Timeout Checklist is good for:**  **Helping to catch/identify potential issues or confusion associated with:** | | | | | |
| --- | --- | --- | --- | --- | --- |
| Group | A patient’s allergies | A patient’s coagulation ability | A patient’s bleeding tendency | Administering the wrong amount of regional anesthetic given a patient’s weight | The type of surgery or procedure to be performed |
| 1 | 4 | 4 | 4 | 4 | 4 |
| 2 | 4 | 4 | 4 | 4 | 5 |
| 3 |  |  |  |  |  |
| 4 | 5 | 5 | 5 | 5 | 5 |
| 5 | 5 | 5 | 5 | 5 | 5 |
| 6 | 4 | 4 | 4 | 4 | 4 |
| 7 | 4 | 4 | 4 | 4 | 4 |
| 8 | 4 | 4 | 4 | 5 | 4 |
| 9 | 5 | 5 | 5 | 5 | 5 |
| 10 | 4 | 3 | 3 | 5 | 5 |
| 11 | 5 | 5 | 5 | 5 | 5 |

1. **Table: After Study Opinions Attending Part 9 Scenarios and Checklist Utility C**

**1 = Strongly Disagree 2 = Disagree 3 = Neutral 4 = Agree 5 = Strongly Agree**

| **I think the Pediatric Regional Anesthesia Timeout Checklist is good for:**  **Helping to catch/identify potential issues or confusion associated with:** | | | | | |
| --- | --- | --- | --- | --- | --- |
| Group | The type of block to be administered | Tracking the dose and timing of other local anesthetics previously administered | Preventing exceeding the maximum allowable local anesthetic for block | Preventing wrong side block | Prevention of Local Anesthetic Systemic Toxicity (LAST) |
| 1 | 5 | 5 | 5 | 5 | 3 |
| 2 | 4 | 4 | 4 | 4 | 4 |
| 3 | 4 | 4 | 4 | 4 | 4 |
| 4 | 4 | 4 | 4 | 5 | 3 |
| 5 | 5 | 5 | 5 | 5 | 5 |
| 6 | 5 | 5 | 5 | 5 | 5 |
| 7 | 5 | 5 | 5 | 5 | 5 |
| 8 | 5 | 5 | 5 | 5 | 5 |
| 9 | 5 | 5 | 5 | 5 | 3 |
| 10 | 4 | 4 | 4 | 4 | 4 |
| 11 | 4 | 5 | 5 | 5 | 3 |

1. **Table: After Study Opinions Resident Part 9 Scenarios and Checklist Utility C**

**1 = Strongly Disagree 2 = Disagree 3 = Neutral 4 = Agree 5 = Strongly Agree**

| **I think the Pediatric Regional Anesthesia Timeout Checklist is good for:**  **Helping to catch/identify potential issues or confusion associated with:** | | | | | |
| --- | --- | --- | --- | --- | --- |
| Group | The type of block to be administered | Tracking the dose and timing of other local anesthetics previously administered | Preventing exceeding the maximum allowable local anesthetic for block | Preventing wrong side block | Prevention of Local Anesthetic Systemic Toxicity (LAST) |
| 1 | 4 | 4 | 4 | 4 | 4 |
| 2 | 5 | 3 | 5 | 5 | 4 |
| 3 | 5 | 5 | 5 | 5 | 5 |
| 4 | 5 | 5 | 5 | 5 | 5 |
| 5 | 5 | 5 | 5 | 5 | 5 |
| 6 | 4 | 4 | 4 | 4 | 4 |
| 7 | 4 | 4 | 4 | 4 | 3 |
| 8 | 4 | 5 | 4 | 4 | 2 |
| 9 | 5 | 5 | 5 | 5 | 5 |
| 10 | 5 | 5 | 5 | 5 | 5 |
| 11 | 5 | 5 | 5 | 5 | 5 |

1. **Table: After Study Opinions Attending Part 9 Scenarios and Checklist Utility D**

**1 = Strongly Disagree 2 = Disagree 3 = Neutral 4 = Agree 5 = Strongly Agree**

| **I think the Pediatric Regional Anesthesia Timeout Checklist is good for:**  **Helping to catch/identify potential issues or confusion associated with:** | | | | |
| --- | --- | --- | --- | --- |
| Group | Identifying when Local Anesthetic Systemic Toxicity (LAST) has occurred | Ensuring that the LAST kit is available | Ensuring that anesthetic for block and related equipment is available and set up |  |
| 1 | 5 | 5 | 5 |  |
| 2 | 4 | 4 | 4 |  |
| 3 | 2 | 3 | 3 |  |
| 4 | 3 | 4 | 4 |  |
| 5 | 5 | 5 | 5 |  |
| 6 | 1 | 4 | 4 |  |
| 7 | 3 | 4 | 5 |  |
| 8 | 3 | 5 | 5 |  |
| 9 | 3 | 5 | 5 |  |
| 10 | 4 | 4 | 4 |  |
| 11 | 2 | 4 | 4 |  |

1. **Table: After Study Opinions Resident Part 9 Scenarios and Checklist Utility D**

**1 = Strongly Disagree 2 = Disagree 3 = Neutral 4 = Agree 5 = Strongly Agree**

|  | | **I think the Pediatric Regional Anesthesia Timeout Checklist is good for:**  **Helping to catch/identify potential issues or confusion associated with:** | | | | |
| --- | --- | --- | --- | --- | --- | --- |
| Group | Identifying when Local Anesthetic Systemic Toxicity (LAST) has occurred | | Ensuring that the LAST kit is available | Ensuring that anesthetic for block and related equipment is available and set up | Additional opinion written on sheet |  |
| 1 | 4 | | 4 | 4 |  |  |
| 2 | 3 | | 5 | 5 |  |  |
| 3 | 5 | | 5 | 5 |  |  |
| 4 | 4 | | 5 | 5 |  |  |
| 5 | 2 | | 5 | 5 | With checklist + additional info, if there was a strong c/I to a block (that I knew), I would not wait until we were to that point in the checklist to bring it up. However, this checklist would help identify any of those contraindications that might have been missed in an earlier preop discussion |  |
| 6 | 4 | | 4 | 4 | [blank] |  |
| 7 | 3 | | 4 | 4 | [blank] |  |
| 8 | 1 | | 4 | 4 | Items “a patient’s coagulation ability” and “A patient’s bleeding tendency” could be combined |  |
| 9 | 5 | | 5 | 5 |  |  |
| 10 | 5 | | 5 | 5 |  |  |
| 11 | 5 | | 5 | 5 |  |  |

1. **Table: Checklist Opinions Attending**

**1 = Strongly Disagree 2 = Disagree 3 = Neutral 4 = Agree 5 = Strongly Agree**

| Group | For normal practice, I do **not** think checklists are very useful. | For response to emergencies in the OR, I do **not** think that checklists are very useful. | A normal checklist for administering regional anesthetics is generally unnecessary. |
| --- | --- | --- | --- |
| 1 | 2 | 2 | 2 |
| 2 | 2 | 1 | 2 |
| 3 | 1 | 1 | 2 |
| 4 | 2 | 2 | 2 |
| 5 | 1 | 1 | 1 |
| 6 | 2 | 2 | 2 |
| 7 | 2 | 1 | 2 |
| 8 | 1 | 1 | 2 |
| 9 | 3 | 3 | 2 |
| 10 | 3 | 5 | 3 |
| 11 | 2 | 1 | 2 |
| Median[IQR] | 0[1,2] |  | 2[2,2] |

1. **Table: Checklist Opinions Resident/Fellow**

**1 = Strongly Disagree 2 = Disagree 3 = Neutral 4 = Agree 5 = Strongly Agree**

| Group | For normal practice, I do **not** think checklists are very useful. | For response to emergencies in the OR, I do **not** think that checklists are very useful. | A normal checklist for administering regional anesthetics is generally unnecessary. |
| --- | --- | --- | --- |
| 1 | 2 | 2 | 2 |
| 2 | 1 | 1 | 2 |
| 3 | 2 | 1 | 1 |
| 4 | 1 | 1 | 1 |
| 5 | 2 | 2 | 3 |
| 6 | 3 | 2 | 3 |
| 7 | 2 | 1 | 2 |
| 8 | 1 | 1 | 2 |
| 9 | 2 | 1 | 2 |
| 10 | 1 | 1 | 2 |
| 11 | 1 | 1 | 1 |
