# Supplementary material for: Evaluation of the Pediatric Regional Anesthesia Time‐Out Checklist: A Simulation Study
Source: Paediatr Anaesth. 2025 Jan 24;35(6):430–8. doi: 10.1111/pan.15069 (PMC12060083; doi:10.1111/pan.15069)
Supplement: Supplementary file 1 — Data S1. [file PAN-35-430-s001.zip › SupplementalMaterial_TrainingForScenarios.pptx]

## Slide 1
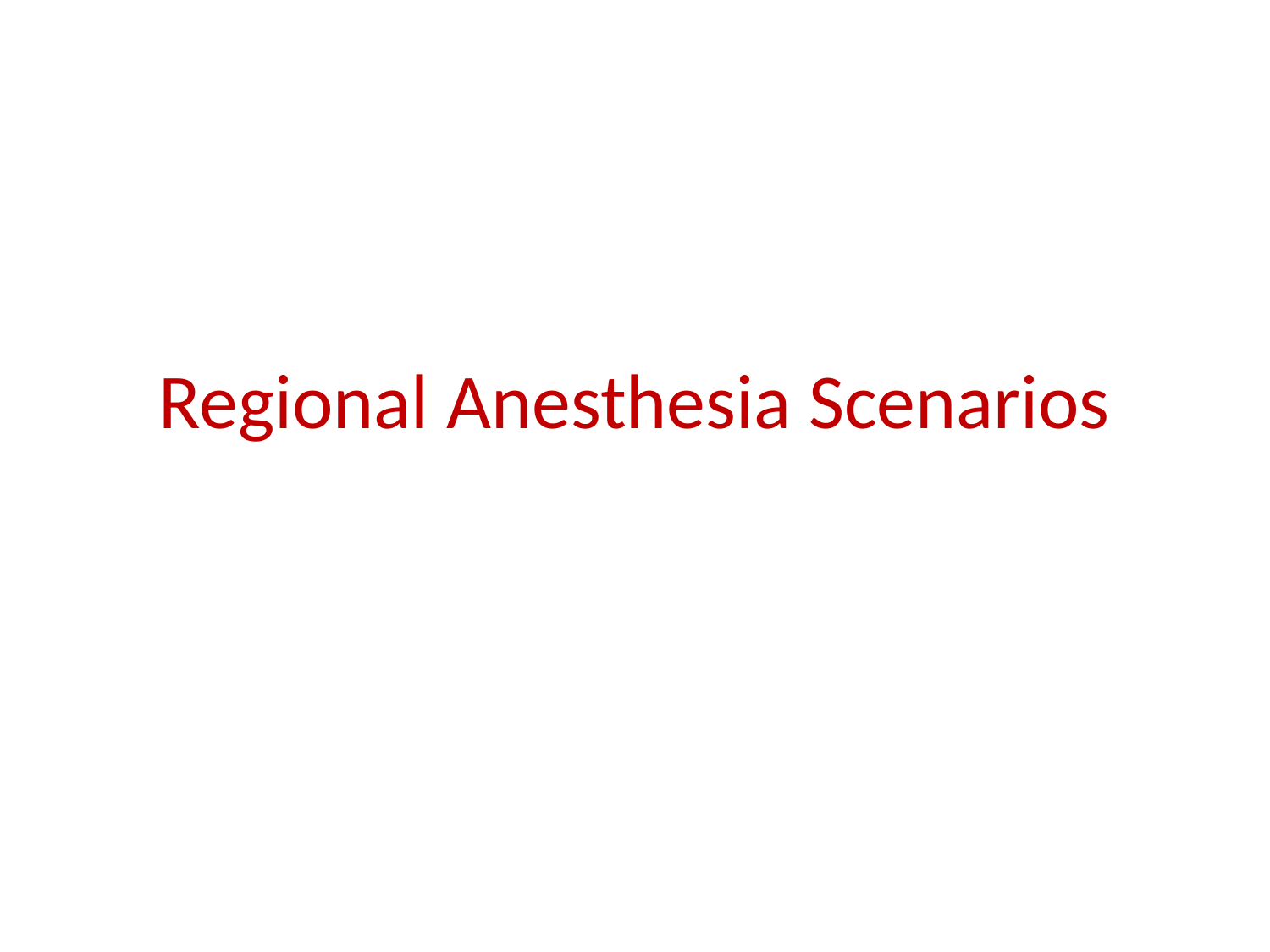

# Regional Anesthesia Scenarios

## Slide 2
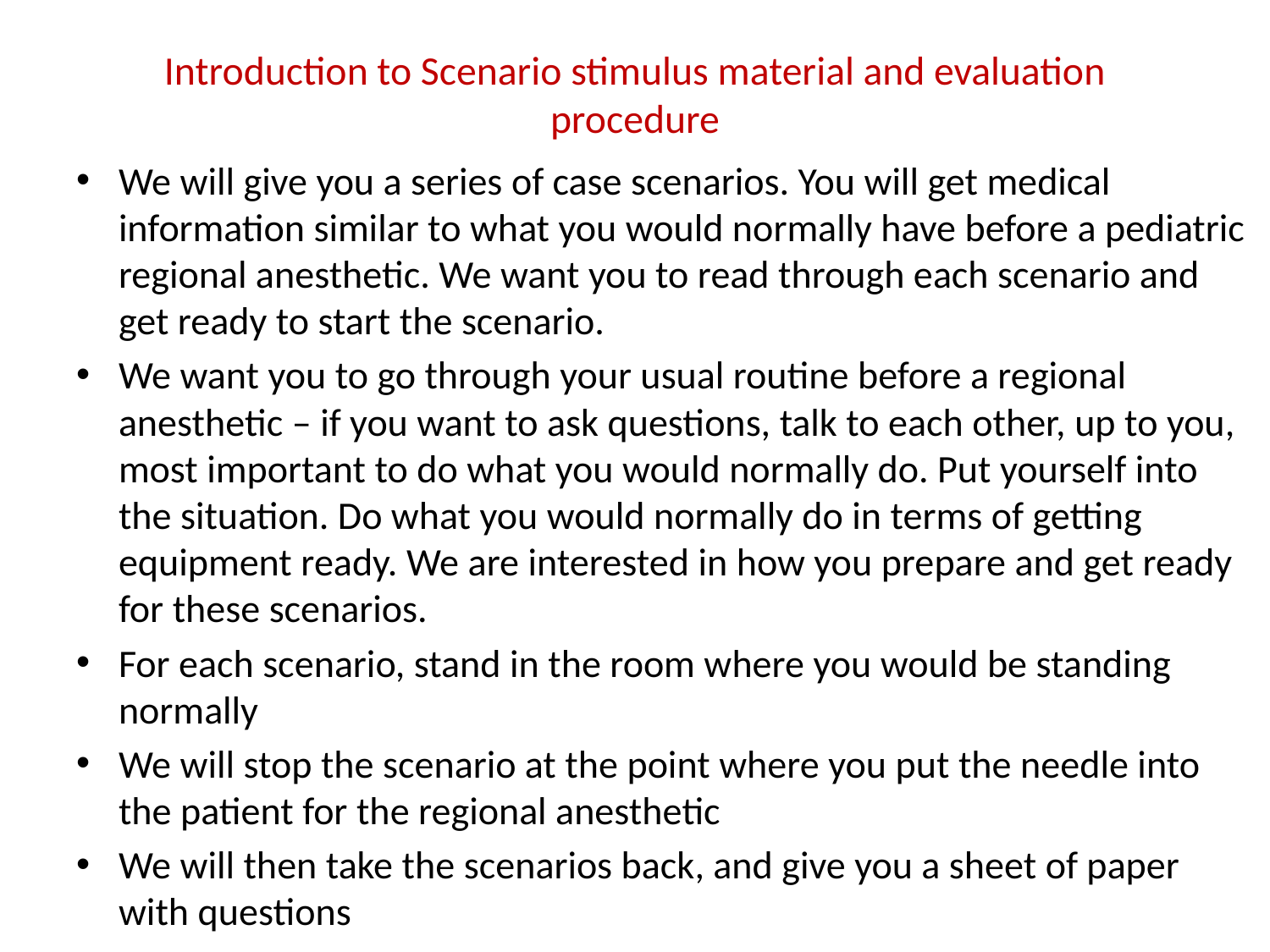

# Introduction to Scenario stimulus material and evaluation procedure
We will give you a series of case scenarios. You will get medical information similar to what you would normally have before a pediatric regional anesthetic. We want you to read through each scenario and get ready to start the scenario.
We want you to go through your usual routine before a regional anesthetic – if you want to ask questions, talk to each other, up to you, most important to do what you would normally do. Put yourself into the situation. Do what you would normally do in terms of getting equipment ready. We are interested in how you prepare and get ready for these scenarios.
For each scenario, stand in the room where you would be standing normally
We will stop the scenario at the point where you put the needle into the patient for the regional anesthetic
We will then take the scenarios back, and give you a sheet of paper with questions
