# Supplementary material for: Evaluation of the Pediatric Regional Anesthesia Time‐Out Checklist: A Simulation Study
Source: Paediatr Anaesth. 2025 Jan 24;35(6):430–8. doi: 10.1111/pan.15069 (PMC12060083; doi:10.1111/pan.15069)
Supplement: Supplementary file 1 — Data S1. [file PAN-35-430-s001.zip › SupplementalMaterials_ORIGINALDATA_Surveys.docx]

**ANESTHESIA ATTENDING: First Half of Study Survey PAGE 1/2**

**THE FOLLOWING QUESTIONS REFER TO THE STUDY SCENARIOS YOU HAVE PARTICIPATED IN SO FAR:**

|  | **Very Unsafe Very Safe** | | | | |
| --- | --- | --- | --- | --- | --- |
| Overall, during the last 6 regional anesthesia scenarios, how safe did I feel that the patients were? | **1** | **2** | **3** | **4** | **5** |
| **Why?** | | | | | |

|  | **Very Poor Very Good** | | | | |
| --- | --- | --- | --- | --- | --- |
| Overall, during the last 6 regional anesthesia scenarios, how good was the teamwork? | **1** | **2** | **3** | **4** | **5** |
| **Why?** | | | | | |

**Demographics and Background Survey**

**1) Current Position:**

____Anesthesia Attending; number of years:________

**2) Board Certification:** ___________________

**Fellowship Training:** ___________________

**Additional Residency Training Besides Anesthesiology:** ___________________

**3) Gender:** ____male ____female

**4) Age:** ________

5**) Attendings and residents: Number of regional blocks administered or supervised in patients less than 21 years old:**

Over the past year, average number/month (circle number):

**0 1-2 2-5 5-10 >10**

**6) Number of years I have been performing regional anesthetics:**

**None 1-2 2-5 5-10 >10**

**ANESTHESIA ATTENDING: First Half of Study Survey PAGE 2/2**

**7) How familiar are you with the other participants in the group?**

**(Number of times worked with previously)**

1. **Anesthesia Resident/Fellow ______ number b) OR Nurse ______ number**

**Background:**

*Please circle the number that best answers the question or most closely reflects your opinion*

*
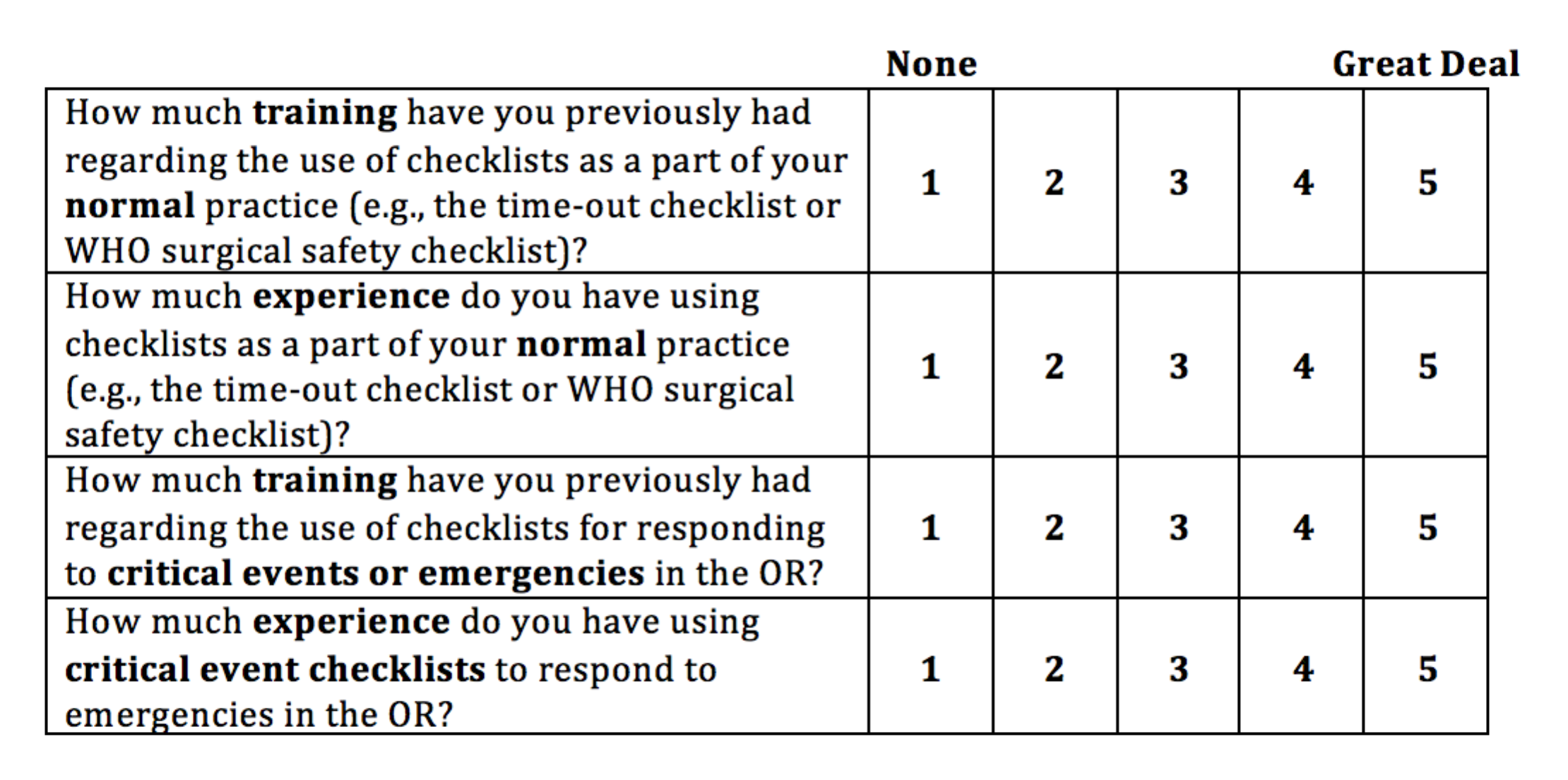
*

|  | **Strongly Disagree** | **Disagree** | **Neutral** | **Agree** | **Strongly**  **Agree** |
| --- | --- | --- | --- | --- | --- |
| For normal practice, I do **not** think checklists are very useful. | **1** | **2** | **3** | **4** | **5** |
| For response to emergencies in the OR, I do **not** think that checklists are very useful. | **1** | **2** | **3** | **4** | **5** |
| A normal checklist for administering regional anesthetics is generally unnecessary. | **1** | **2** | **3** | **4** | **5** |

**ANESTHESIA RESIDENT/FELLOW: First Half of Study Survey PAGE 1/2**

**THE FOLLOWING QUESTIONS REFER TO THE STUDY SCENARIOS YOU HAVE PARTICIPATED IN SO FAR:**

|  | **Very Unsafe Very Safe** | | | | |
| --- | --- | --- | --- | --- | --- |
| Overall, during the last 6 regional anesthesia scenarios, how safe did I feel that the patients were? | **1** | **2** | **3** | **4** | **5** |
| **Why?** | | | | | |

|  | **Very Poor Very Good** | | | | |
| --- | --- | --- | --- | --- | --- |
| Overall, during the last 6 regional anesthesia scenarios, how good was the teamwork? | **1** | **2** | **3** | **4** | **5** |
| **Why?** | | | | | |

**Demographics and Background Survey**

**Current Position:**

____Anesthesia Resident: ____1^st^ year ____2^nd^ year ____3^rd^ year ____4^th^ year ___Fellow

**Fellowship Training:** ___________________

**Additional Residency Training Besides Anesthesiology:** ___________________

**Gender:** ____male ____female

**Age:** ________

**Residents and Fellows: Number of regional blocks administered or supervised in patients less than 21 years old:**

**Over the past year, average number/month (circle number):**

**0 1-2 2-5 5-10 >10**

**Number of years I have been performing regional anesthetics:**

**None 1-2 2-5 5-10 >10**

**ANESTHESIA RESIDENT/FELLOW: First Half of Study Survey PAGE 2/2**

**6) How familiar are you with the other participants in the group?**

**(Number of times worked with previously)**

a) Anesthesia Attending ______ number b) OR Nurse ______ number

**Background:**

*Please circle the number that best answers the question or most closely reflects your opinion*

*
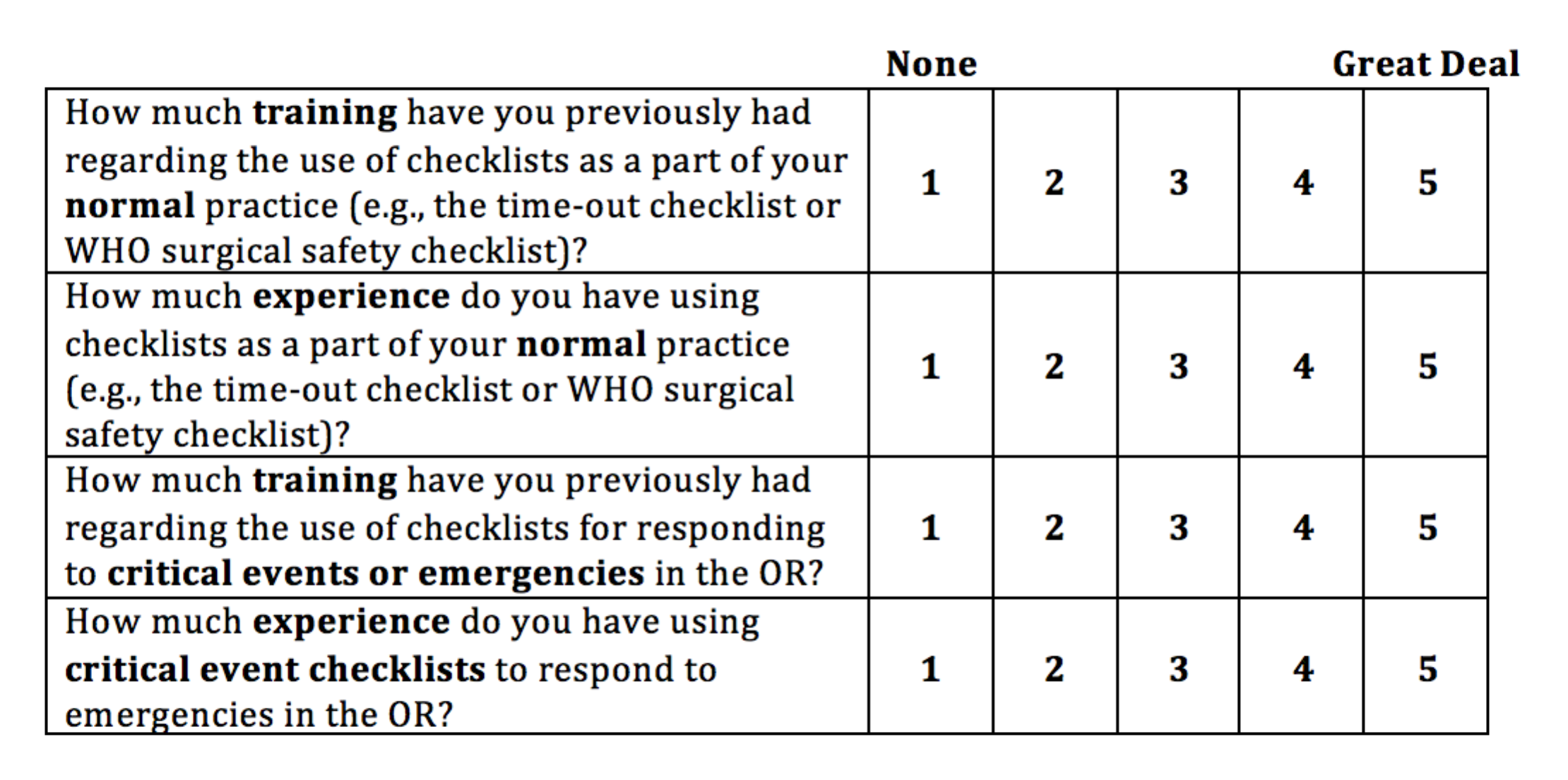
*

|  | **Strongly Disagree** | **Disagree** | **Neutral** | **Agree** | **Strongly**  **Agree** |
| --- | --- | --- | --- | --- | --- |
| For normal practice, I do **not** think checklists are very useful. | **1** | **2** | **3** | **4** | **5** |
| For response to emergencies in the OR, I do **not** think that checklists are very useful. | **1** | **2** | **3** | **4** | **5** |
| A normal checklist for administering regional anesthetics is generally unnecessary. | **1** | **2** | **3** | **4** | **5** |

**AFTER STUDY SURVEY ANESTHESIA ATTENDING PAGE 1/4**

**
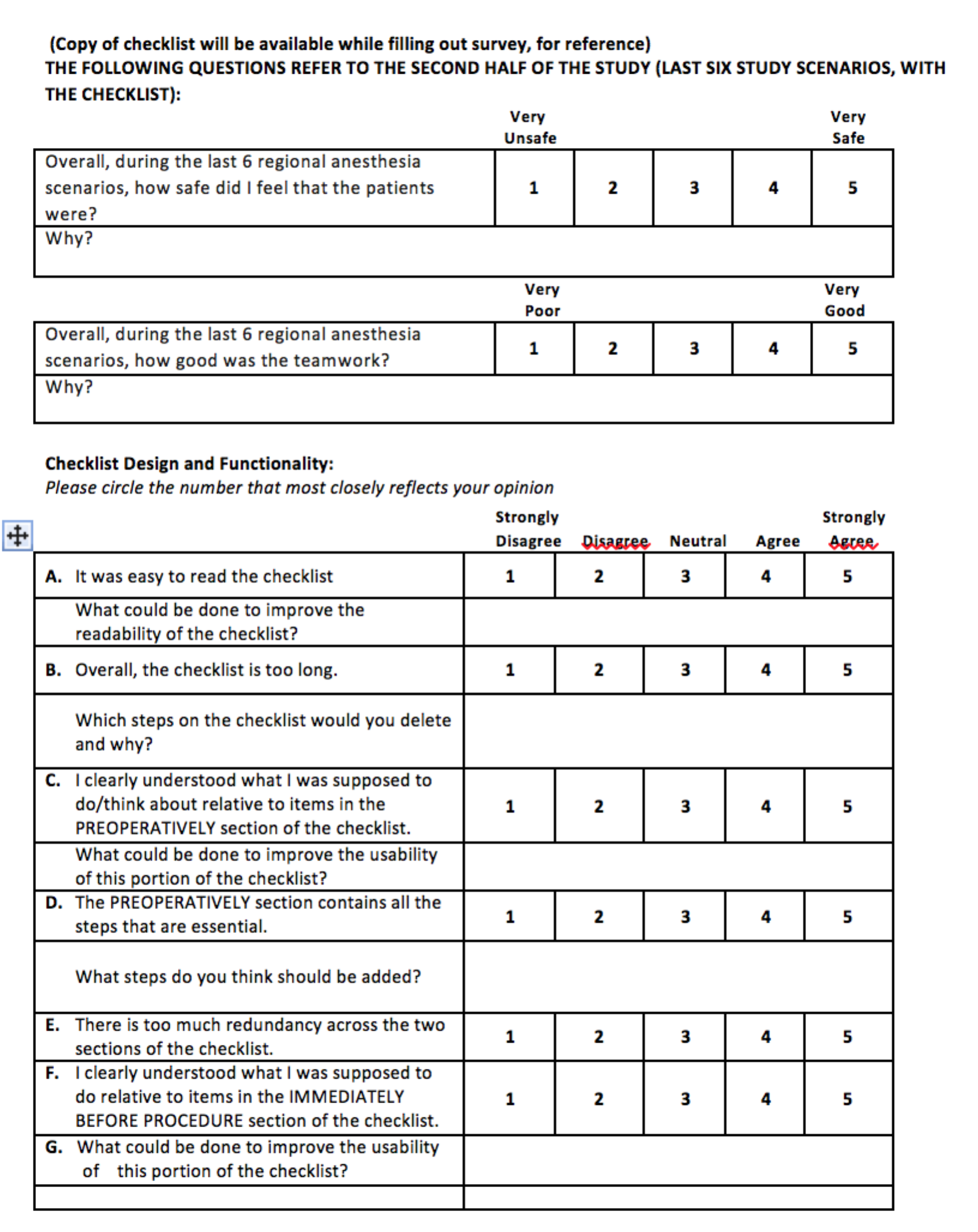
**

**AFTER STUDY SURVEY ANESTHESIA ATTENDING PAGE 2/4**

**
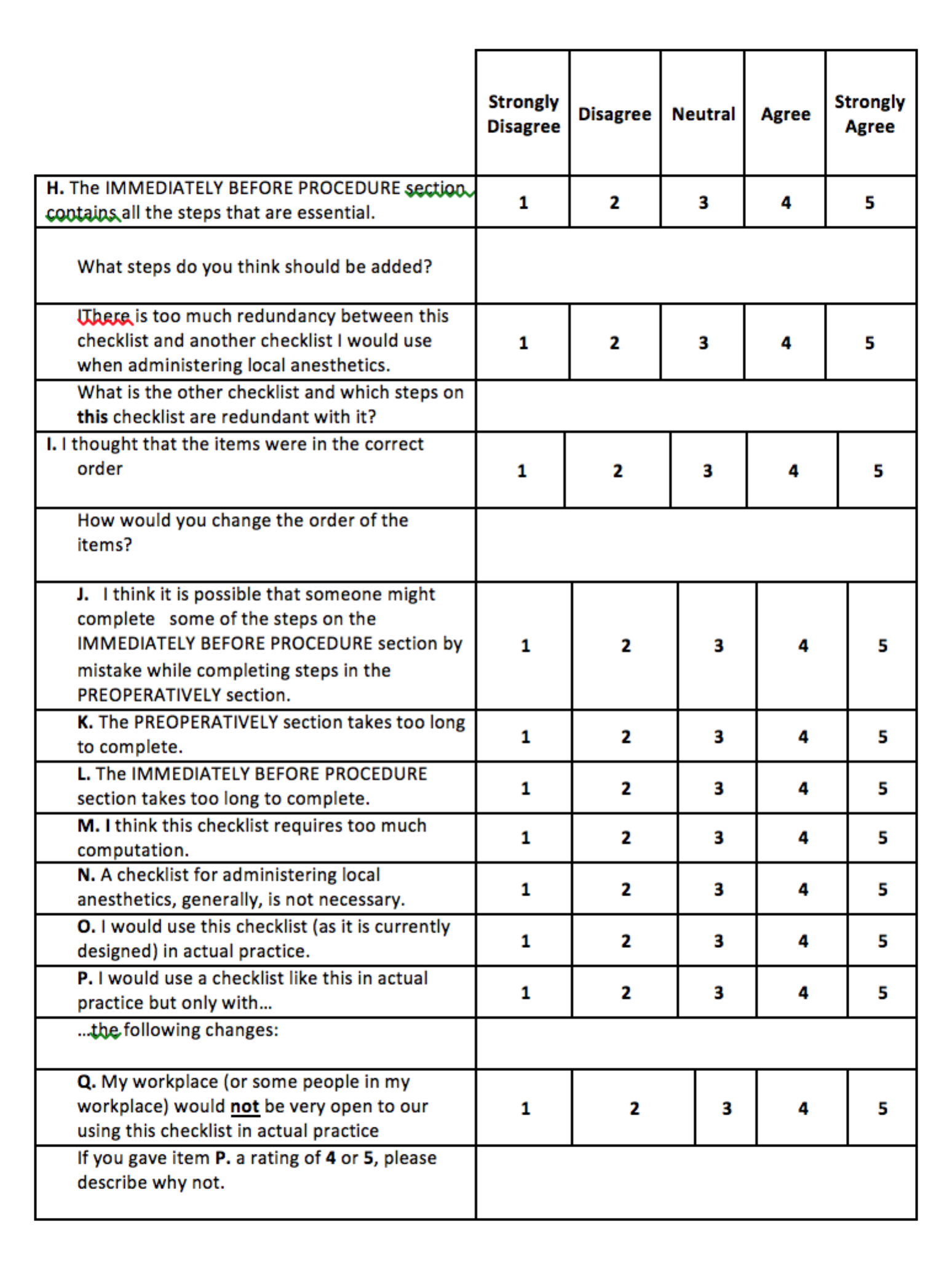
**

**AFTER STUDY SURVEY ANESTHESIA ATTENDING PAGE 3/4**

**
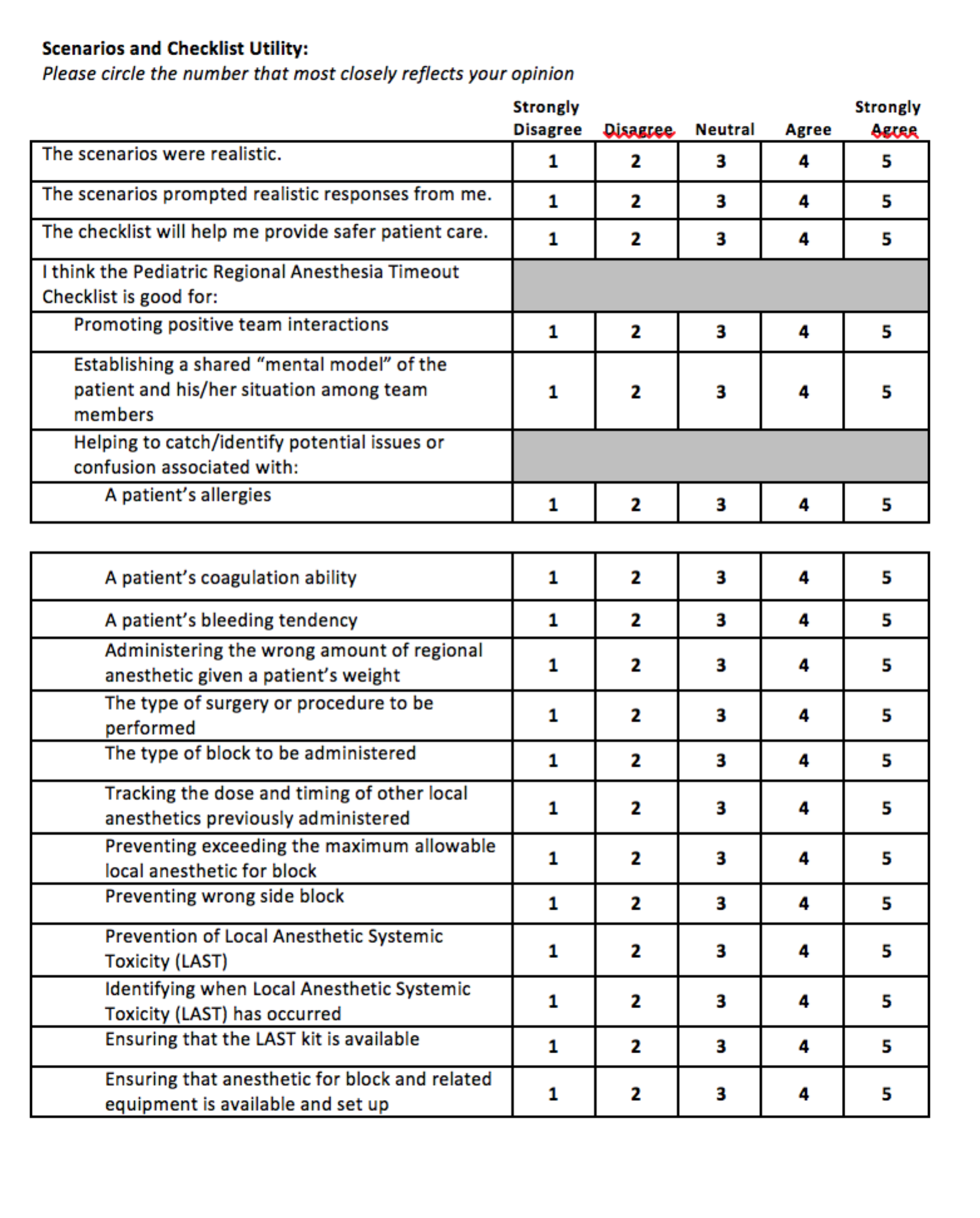
**

**AFTER STUDY SURVEY ANESTHESIA ATTENDING PAGE 4/4**

**
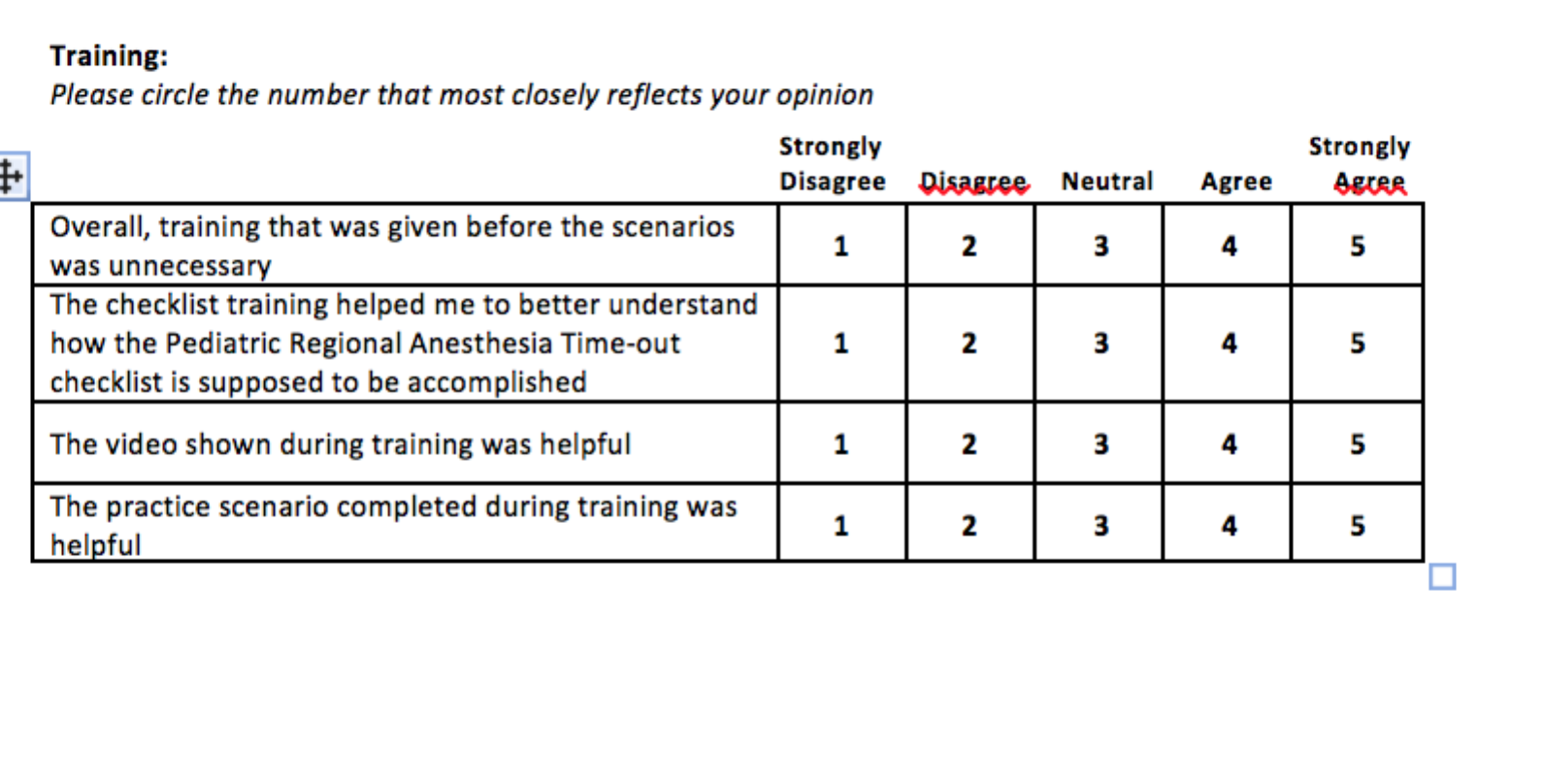
**

**AFTER STUDY SURVEY ANESTHESIA RESIDENT/FELLOW PAGE 1/4**

**
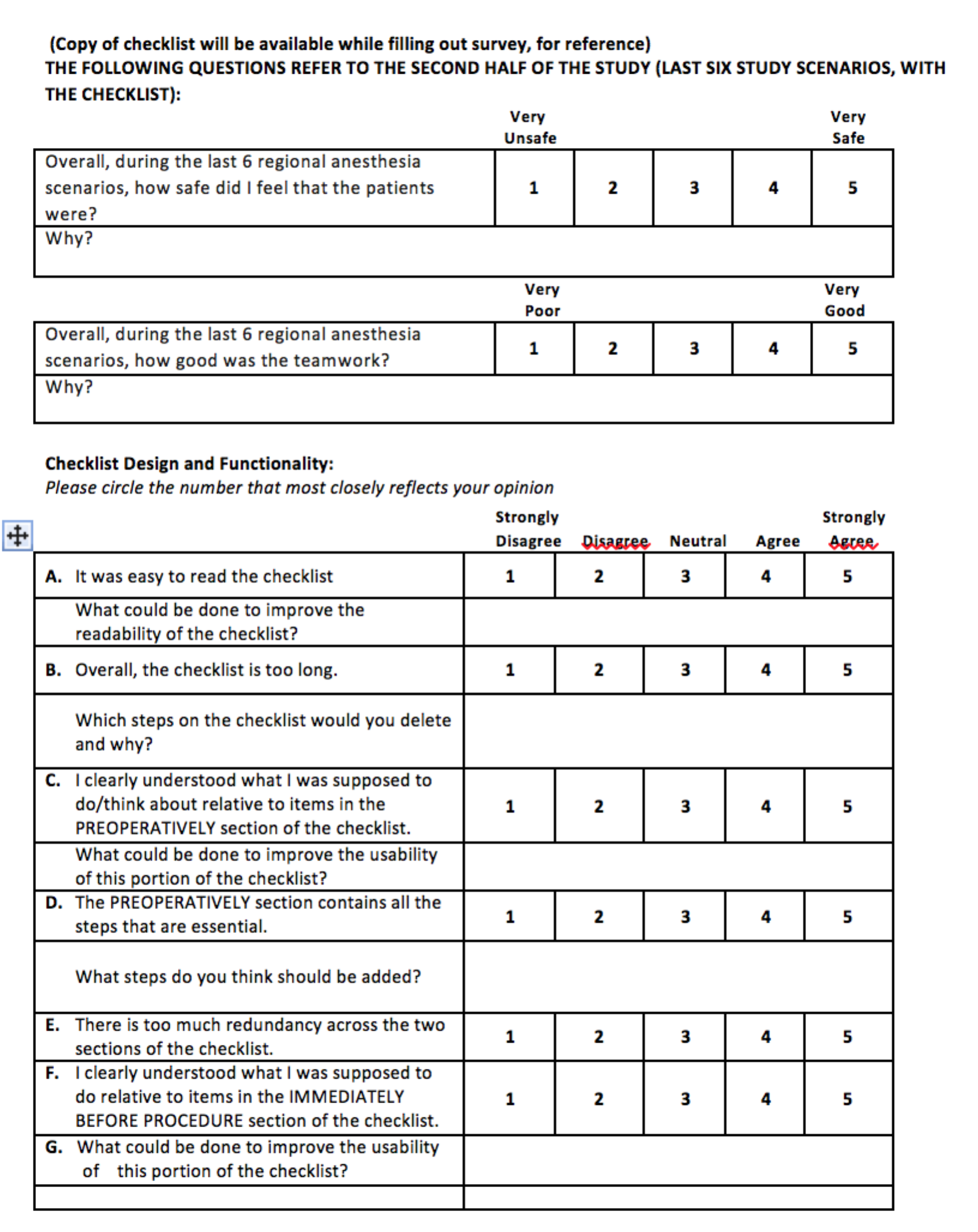
**

**AFTER STUDY SURVEY ANESTHESIA RESIDENT PAGE 2/4**

**
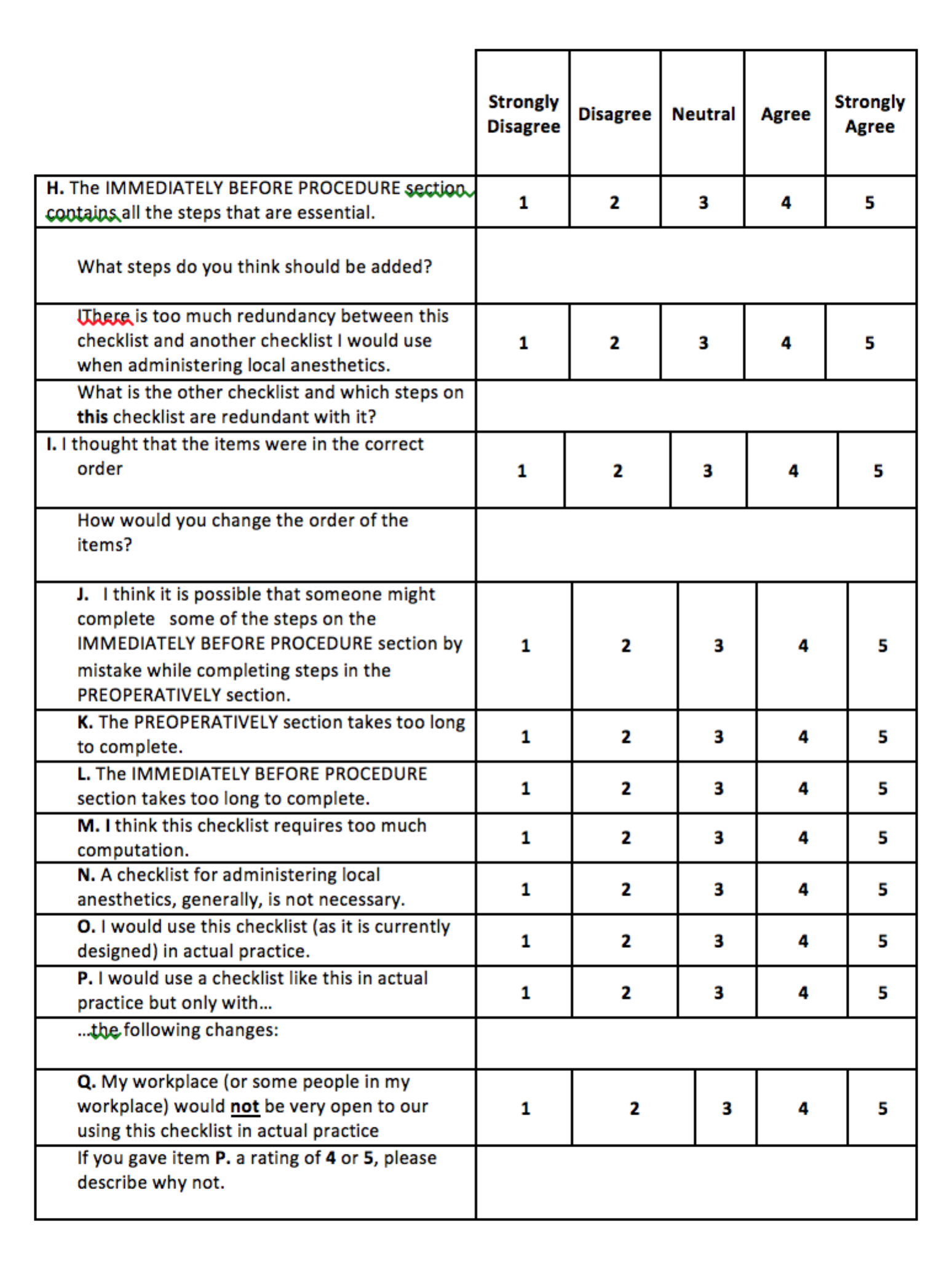
**

**AFTER STUDY SURVEY ANESTHESIA RESIDENT PAGE 3/4**

**
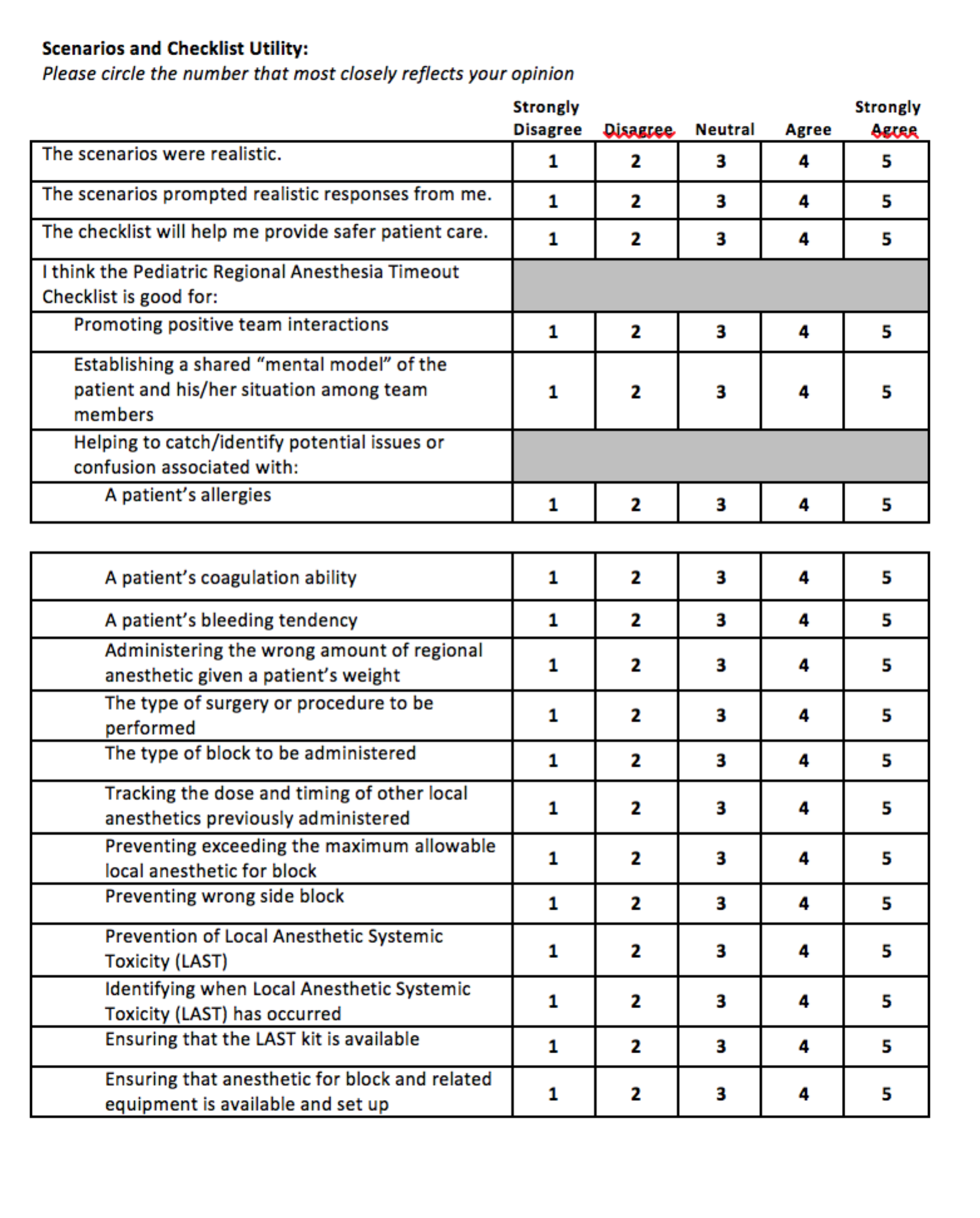
**

**AFTER STUDY SURVEY ANESTHESIA RESIDENT PAGE 4/4**

**
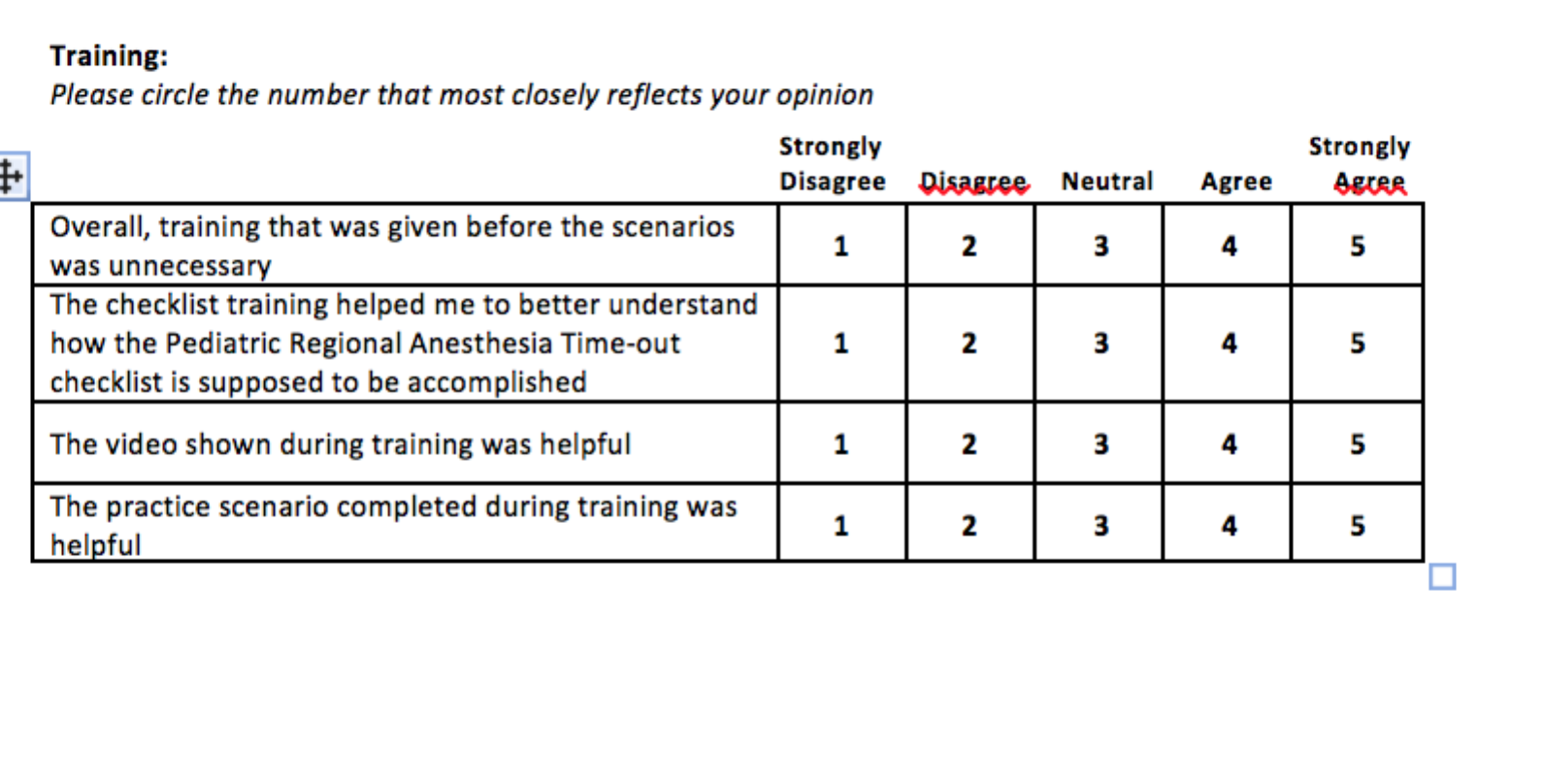
**
